# Guided Tissue and Bone Regeneration Membranes: A Review of Biomaterials and Techniques for Periodontal Treatments

**DOI:** 10.3390/polym15163355

**Published:** 2023-08-10

**Authors:** Ali M. Alqahtani, Robert Moorehead, Ilida Ortega Asencio

**Affiliations:** 1 Department of Restorative Dental Sciences, College of Dentistry, King Khalid University, Al Fara, Abha 62223, Saudi Arabia; aqahtani@kku.edu.sa; 2 The Department of Materials Science and Engineering, The University of Sheffield, Sir Robert Hadfield Building, Sheffield S1 3JD, UK; r.moorehead@sheffield.ac.uk; 3 The School of Clinical Dentistry, The University of Sheffield, 19 Claremont Crescent, Sheffield S10 2TA, UK

**Keywords:** dental biomaterials, guided bone regeneration, guided tissue regeneration, tissue engineering, biocompatible polymers, membranes, scaffolds

## Abstract

This comprehensive review provides an in-depth analysis of the use of biomaterials in the processes of guided tissue and bone regeneration, and their indispensable role in dental therapeutic interventions. These interventions serve the critical function of restoring both structural integrity and functionality to the dentition that has been lost or damaged. The basis for this review is laid through the exploration of various relevant scientific databases such as Scopus, PubMed, Web of science and MEDLINE. From a meticulous selection, relevant literature was chosen. This review commences by examining the different types of membranes used in guided bone regeneration procedures and the spectrum of biomaterials employed in these operations. It then explores the manufacturing technologies for the scaffold, delving into their significant impact on tissue and bone regenerations. At the core of this review is the method of guided bone regeneration, which is a crucial technique for counteracting bone loss induced by tooth extraction or periodontal disease. The discussion advances by underscoring the latest innovations and strategies in the field of tissue regeneration. One key observation is the critical role that membranes play in guided reconstruction; they serve as a barrier, preventing the entry of non-ossifying cells, thereby promoting the successful growth and regeneration of bone and tissue. By reviewing the existing literature on biomaterials, membranes, and scaffold manufacturing technologies, this paper illustrates the vast potential for innovation and growth within the field of dental therapeutic interventions, particularly in guided tissue and bone regeneration.

## 1. Introduction

The field of periodontology has seen considerable advancements in recent years, with a keen focus on therapeutic strategies for restoring periodontal lesions and regenerating lost jawbone through cellular proliferation. Central to this endeavor is the availability of a substantial volume of hard bone tissue, the foundation for successful implant treatments [1].

The complex architecture of a healthy periodontium, with its multilayered structure and dynamic interplay of cells, tissues, and molecular factors, is fundamental to oral health. However, the periodontium can fall prey to a range of pathological conditions, leading to tooth loss and degenerative changes [2]. These conditions necessitate a host of diverse treatment modalities, from traditional periodontal therapies to more contemporary, regenerative procedures.

In the realm of regenerative therapies, guided bone regeneration (GBR) and guided tissue regeneration (GTR) have garnered significant attention. Primarily, GBR focuses on the regeneration of alveolar bone in edentulous regions, while GTR is tasked with repairing compromised periodontal tissues [3,4]. Both these techniques leverage the utility of a porous polymer membrane to physically impede the infiltration of undesirable tissues and cells into the lesion site [4]. This strategy aids in fostering an environment conducive for the proliferation of required cells [5].

GBR and GTR techniques play a wide array of roles, with GBR involved in maintaining and enhancing the alveolar ridge, correcting implant contractions or fenestrations, and promoting bone regeneration around implants [6]. Conversely, GTR is engaged in the regeneration of the periodontal ligament (PDL), bone, and cementum in proximity to the tooth [7].

However, successful bone and tissue regeneration are not merely reliant on the prevention of undesirable cell infiltration; they also demand the presence of osteogenic cells, alongside osteoconductive and osteoinductive materials [8]. Membranes, integral to GBR and GTR, should exhibit excellent biocompatibility and extended functional stability. They are also expected to ensure the spatial and biomechanical stability of the lesion site by filtering disruptive cells and tissues and protecting the emergent tissue [9].

Furthermore, these membranes are classified into several types based on their composition and bioactivity, such as bioabsorbable, non-resorbable, and metal and inorganic compound membranes [10]. In the latter part of this review, the pivotal role of scaffold manufacturing technologies, as a subset of these membrane technologies, in bone and tissue regenerations will be thoroughly discussed. This provides a unique vantage point to appreciate the wide spectrum of cutting-edge strategies that have been developed for the regeneration of bone and tissue in the periodontal context.

## 2. Materials and Methods

To conduct this comprehensive review, we sourced primary data from several established scientific databases, including Scopus, PubMed, and MEDLINE. Our objective was to unearth the most relevant and impactful literature relating to the use of biomaterials in guided tissue and bone regeneration procedures.

Our search strategy was developed with a focus on several key terms and phrases pertinent to our study. These include “biocompatible materials”, “membrane”, “bone regeneration”, “tissue regeneration”, and “dental biomaterials”. We meticulously scanned all abstracts yielded by these search terms and selected full-text articles that aligned most significantly with our study’s aims and objectives.

The review process involved a rigorous methodological approach. All selected articles underwent detailed evaluation, where data relating to the membrane types and range of biomaterials used for tissue and bone regeneration were extracted and scrutinized. In addition, we paid particular attention to articles that discussed the role of scaffold manufacturing technologies in these regenerative procedures.

Furthermore, we performed a narrative synthesis of the data obtained. The synthesis was aimed at providing a comprehensive overview of the current understanding and advancements in the use of biomaterials for guided tissue and bone regeneration, as well as the role of membranes in preventing the ingress of non-ossifying cells.

The exclusion criteria applied in the literature review process helped to maintain the focus and relevance of this study. Articles that did not focus on dental tissue and bone regeneration, used non-biocompatible materials, or did not discuss the use of scaffold manufacturing technologies were excluded from the review. Additionally, articles published in languages other than English were not considered.

This methodical approach ensured the selected literature was of high quality and relevant to our study, thereby supporting a more accurate and comprehensive review of the subject matter.

## 3. Results

Current periodontal treatment approaches are targeted at minimizing and/or removing inflamed tissues induced by bacterial plaque, repairing deficiencies or structural abnormalities, and regenerating new tissues in the region of lost tissues [11,12,13]. Various methods mentioned previously are only able to stop the progress of the problem, but are unable to reverse the damage or replace the lost tissue [14]. Bone grafting, enamel matrix derivative (EMD) and guided regeneration therapy are now used in the development of tissues that have been infected by periodontal diseases. To a certain degree, the overall structure and function of the damaged tissue can be restored [13].

### 3.1. Historical Viewpoint on Approaches to Periodontal Regeneration

**Figure 1:**
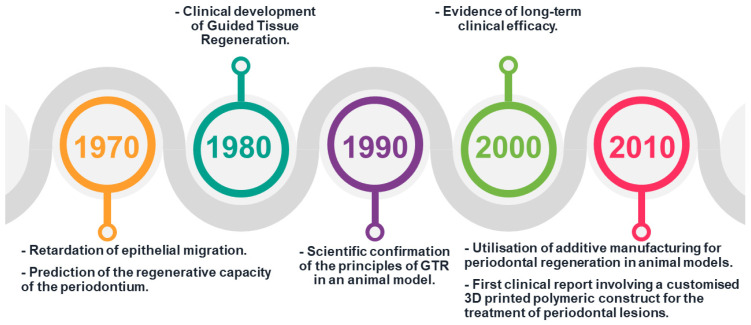
Timeline of periodontal regeneration approaches: from the original idea involving a free palatal graft for inhibiting epithelial migration to the most current developments involving additively engineered polymeric multiphasic scaffolds for periodontal tissue engineering.

The concept of placing a physical barrier along the tooth root surface after periodontal surgery to prevent epithelial downgrowth was first proposed in the 1970s [15] (Figure 1).

Indeed, physicians had previously hypothesized that the collapse of gingival tissues into periodontal defects seriously impeded bone resorption [16,17]. Several early experiments suggested the placing of a harvested free palatal graft over the periodontal defect in order to delay or at least obstruct the downgrowth of epithelium around the tooth root surface [15]. Another popular procedure included the insertion of bone grafts (allogenic, autologous, or synthetic) obtained from the patient inside the periodontal defect to regenerate the missing bone [18]. However, neither of these methods is successful for periodontal recovery, and only periodontal healing is observed in the context of a fresh junctional epithelium. After researching the clinical and laboratory evidence, it can be hypothesized that the lack of compartmentalization between the periodontal defect and the underlying soft tissue was the cause of low regeneration rates. The problem of selective periodontal defect repopulation by tissues capable of fostering periodontal regeneration was presented in a series of pioneering papers by Nyman et al., which contributed to the development of the principle of guided tissue regeneration (GTR) [15,19].

#### 3.1.1. Bone Graft Procedure

**Figure 2:**
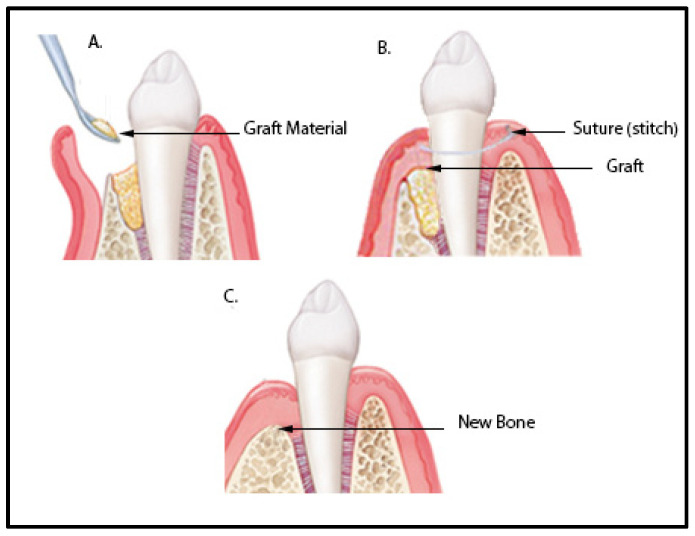
A schematic diagram of the management of periodontal defects by a bone graft technique. (**A**) Placing the graft. First, a gum flap is created. Growth factors may then be applied to the root. Graft material is packed into the area where bone was lost. (**B**) Closing up. The gum is closed and sewn together. (**C**) After the area heals. Stitches dissolve or are removed.

Bone grafts have been an option for a long time to successfully deal with the effects of periodontal disease, such as bone loss and damage. A bone graft is meant to fill the space that originated from the damaged tissue with a material that possesses certain qualities and characteristics (Figure 2). There are several types currently available, such as allografts, xenografts, alloplastic, and autograft materials. These kinds of graft materials are able to facilitate natural osseous repair through some mechanisms that have already been properly characterized [18,20,21]:

- Osteogenesis: The graft possesses cells that function as seeds for the continuous growth of the tissue by forming a bone matrix.
- Osteoinduction: The graft can release factors and biochemical signals that stimulate the formation of new bone by cells.
- Osteoconduction: The graft works as a scaffold on which the host bone develops.

Diverse graft materials can be categorized into four general types that are shown in Table 1.

In the past, research publications have reported that at least 3.0 mm of bone height can be acquired, regardless of which material the graft is made of [21,22]. Probably the best option for osteogenesis, osteoconduction, and osteoinduction is autologous bone since it shares all of its properties with the surrounding bone. In this type, a part of the bone structure is extracted from a normal and un-damaged area of the patient who is getting the graft. The structure that is compatible and inherent to this type makes it very advantageous since it has the same vital bone structures in critical regions and includes nutrients, proteins, and cells as those found in the affected site. However, the autoimmune grafts come with some disadvantages, such as increasing the patient’s pain at the same site of the excision. In addition, only small amounts of bone can be extracted without incurring permanent damage to the patient.

Due to these drawbacks, several xenografts, bone grafts, as well as allogeneic materials have been designed and approved for commercial use [23]. It is important to note that clinicians must take into account the risks associated with these materials, such as infection, resorption, and immune responses. Currently, two types of allografts are available: freeze-dried bone allograft (FDBA) and demineralized freeze-dried bone allograft (DFDBA). These grafts are pre-treated chemically; they keep their osteoinductive capabilities due to the conservation of certain proteins, such as BMPs and TGF-s. These proteins work as powerful growth stimulants and induce the mobilization of cells from the mesenchyme into the implant. The overall process of demineralization enhances the excretion of these highly important factors into the extracellular medium [21,24].

The grafts that are extracted from animals, mainly farm cattle, are called xenografts. They go through chemical treatment in order to remove their inherent antigens, in order to avoid the human body’s natural immune response. The greatest advantage in using these products lies in the fact that the general structure of the graft can be maintained, and this is because of the prolonged amounts of time required for these materials to be resorbed. These materials are capable of osteoconductivity and little else. However, the overall safeness and efficacy of them have been proved to be clinically relevant [21,25].

Due to the risks related to these types of grafts, scientists have also conducted investigations into the use of synthetic alternatives, which include composite grafts, polymer and inorganic materials, to repair osseous tissue [26]. In particular, materials such as hydroxyapatite and β-tricalcium phosphate (β-TCP) have become of increasing clinical importance when studying periodontal diseases. Furthermore, even though synthetic materials lack osteogenic and osteoconductive capabilities, their osteoconductive properties make them excellent options for bone regeneration. Recently, in a study by Schmidlin et al. (2013), it was found that polylactide-coated TCP was sufficient to repair problems in the rabbit’s bone structure, all of this while retaining its biocompatibility [27].

Certain products of natural origin, such as coral, have been shown to be able to be used as bone grafts due to their similarity to human bone structure [28]. Similarly, to synthetic materials, numerous natural products have been proven safe for human use and are very cost-efficient. However, they are still only osteoconductive [21].

#### 3.1.2. Guided Regeneration Therapy

The key aim of periodontal regeneration is to establish new cementum with periodontal ligament (PDL) fiber attached to the alveolar bone and promote new bone growth. Currently, there are two surgical approaches that have been used for regenerating periodontal tissues. These are guided tissue regeneration (GTR) and guided bone regeneration (GBR) [12,29,30]. The concept of guided tissue/bone regeneration began in the late 80s and was developed by Nyman et al. (1990) based on Melcher’s theory. This theory hypotheses that when cells with regenerative capabilities are associated with damaged tissues, they can actually be used to aid in the regeneration of that tissue [19]. The hypothesis has been successfully proven in various animal experiments, and the principle of guided tissue regeneration (GTR) has been confirmed [12,15,29].

GTR refers to the procedure of regenerating periodontal tissue through the use of an occlusive barrier membrane between gingival (epithelial) and alveolar bone/PDL tissue. In this operation, an occlusive membrane will be inserted onto the surgical site in order to inhibit the migration of connective and epithelial tissue through the surgical site [12,15,29]. Progenitor cells that existed in the lining of the residual periodontal ligament, corresponding alveolar bone, or blood can then re-colonize the root region and divide into new periodontal supporting components [29]. The guided bone regeneration (GBR) approach is often used to repair defective alveolar ridges before or in conjunction with the placement of a dental implant at extraction sites. In GBR, a bone defect is protected by a membrane to stop fiber tissue intrusion into the site of the graft and to promote the development of a new bone. Intrabony abnormalities and furcations are also treated with GBR [12,15,29].

**Figure 3:**
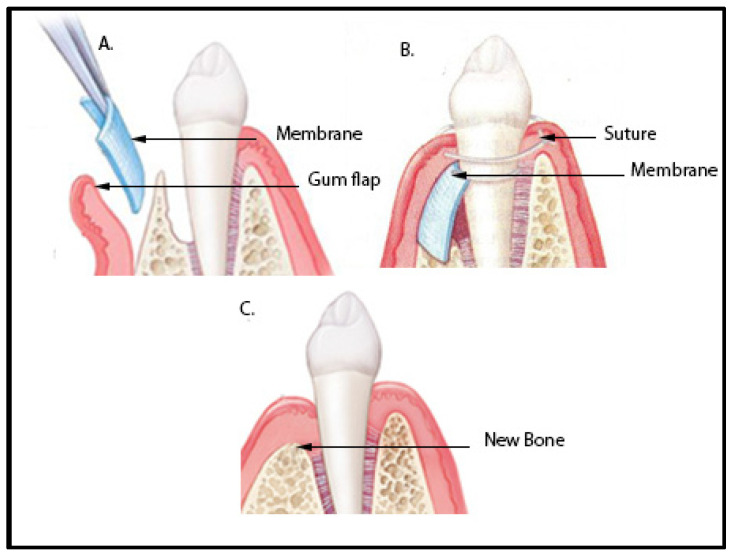
Schematic of GTR is a technique used to repair periodontal defects. (**A**) The gum is opened with a procedure known as a flap. Then, a membrane (with or without the bone graft material) is placed over the damaged bone. (**B**) Closing up. The gum is closed and sewn together. (**C**) After the area heals. Stitches dissolve or are removed.

In order to promote the development of healthy bone structures surrounding bone defects, GBR permeable membranes can be utilized (Figure 3). GBR membranes can also be used in order to preserve the socket area that may be around the tooth due to the presence of periodontal disease, and can even be used to regenerate bone structure at the tooth site after it has been misplaced or extracted [31].

The efficacy of this therapeutic approach was later verified by Gottlow et al., who effectively extended it to a large group of patients [32,33]. Subsequently, the theoretical and biological basis of GBR has been confirmed during the last three decades in several studies [34], and the effectiveness of the procedure has been shown in a multitude of clinical trials [35] and recorded in comprehensive reviews [15,36].

Guided regeneration has been shown to have many benefits when it comes to tissue regeneration over more conventional surgery approaches such as open-flap debridement, which is usually used to address intrabony defects and mild to moderate furcations [15,36].

The concept behind bone augmentation protocols mainly relates to the enhancement in function and aesthetics and can also be used to improve the functions of fixed dental prosthetics, including dental implants and fixed dentures. This procedure greatly helps in correcting contour deficiencies associated with the replacement of artificial teeth as well as offering a friendlier solution to altered speech patterns due to the uncovered areas frequently left between gingival tissues and the restoration [15,37].

#### 3.1.3. Biologic Principles for Guided Regeneration Therapy

In order to successfully develop an engineered tissue, the following essential elements are required: properly defined levels and patterns of regulatory signals; an abundance of progenitor cells; a sufficient blood supply; and an appropriate biomaterial scaffold [38]. Whereas cells serve as the processing facility for the formation of newly formed tissue and the differentiation of cells. Cells require external stimuli in order to stimulate growth and matrix synthesis. These can be provided by growth factors or morphogens. New vascular networks are promoted as a result of angiogenic signals, which supply nutrients for tissue growth and maintenance. The three-dimensional architecture of scaffolds aids in directing cell regeneration [11,15].

The healing process for periodontal surgery wounds follows the same three stages as any other incisional wound. Initially, a fibrin clot is shaped along the flap’s margin and the root surface. Then, a connective tissue matrix attached to the root surface takes the place of the fibrin clot [39]. By keeping the fibrin attached, a new connective tissue bond can form on the root’s outer surface. However, a long-bonding epithelial connection forms if the limit of the fibrin clot’s tensile strength is surpassed [15,40,41].

In general, the method of periodontal healing is more complicated than other wounds by considerations such as the involvement of various specific cell types and complexity of attachments; avascular root surfaces; different microbial flora; and stromal-cellular interfaces [42]. The first biological reaction that happens after the installation of the barrier membrane is the action of the tissue–membrane interface to absorb the plasma protein. Hence, the related growth factors and progenitor cells, which play an important role in tissue repair, are attracted to the surface of the membrane with the help of proteins [43]. In order to provide nourishment to the new tissue in the barrier membrane, which protects defects, much of the vascular supply comes from blood vessels that originate in the marrow [44]. This further explains why it is vital to plan multiple perforations in cortical bone (also called intra-marrow penetration), as this assists in the production of an excess of angiogenic and osteogenic cells as a means of creating new blood vessels and constructing new bone tissue. This serves two objectives: first, it induces bleeding or blood clotting around the grafts in order to induce bone formation around the grafts, and second, it increases the number of factors that raise the likelihood of bone growth [15,37,41].

There is also controversy about the effector cells in periodontal regeneration. Some reports indicate that PDL cells have the potential to behave as osteoblasts or cementoblasts when they are supplied with growth factors and allowed to proliferate. Other data indicate that PDL cells have the ability to regulate mineral formation, so this will help avoid ankylosis when undergoing regeneration [45]. In other studies, PDL cells in vivo and in vitro have been reported to exhibit minimal osteoblastic properties [42,45]. However, other studies argue that osteoblasts, and not PDL cells, are responsible for generating cementum-like material [17,46,47]. Such variations can be attributable to PDL cell heterogeneity, differing study designs, and/or loss of cell properties defined in vitro research. In summary, the majority of evidence points to PDL cells as the main source. Some reports also point to bone cells as the origin of regenerative cells [15,41,48].

### 3.2. Requirements of GTR/GBR Membranes

GTR and GBR membranes need to fulfil specific requirements to be most effective and successful [49]. These requirements can be summarized as follows:

- Cell exclusion: A growth guide membrane can be used to separate several types of unwanted tissue (e.g., epithelial cells) as well as get access to the site of interest [50].
- Framework: A more rigid framework is frequently necessary when clinical cases need more space maintenance in order to prevent membrane compression into the defect site. Bone grafts can provide this support [49,51].
- Porosity: In order to achieve appropriate cell growth and proliferate, the cells must have an underlying high-pore structure [9].
- Degradation: It is important to provide a degradation profile that suits the tissue regeneration, which takes approximately four to six weeks. Ideally, the membrane should fully degrade after it fulfils its purpose without leaving any residual materials [52].
- Stabilization: To prevent mechanical disturbances from the outside and overhanging of flap movement during the process of healing. Mini screws or sutures, can be used to keep the membrane in place [49].
- Clinical manageability: The membrane and the barrier need to possess physical characteristics that enable their handling by the clinician [53].
- Biocompatibility: Inflammation should be avoided at all costs in order to avoid increased morbidity and costs [49,54].

Owing to the large number of scaffolds that can be made from a wide range of materials, these materials have differing degrees of degradation and integrity. These scaffolds can often induce immune reactions in the hosts [55]. The following section discusses commercial membranes, whether non-absorbable or absorbable, in guided regeneration therapy.

### 3.3. Types of Commercial Membranes Used in Guided Regeneration Therapy

Barriers used in bone/tissue regeneration procedures have varying degrees of deterioration and properties and, therefore, have generally been divided into resorbable or non-absorbable membranes. Gottlow, (1993) was the first to divide these membranes into two generations depending on when they were created and developed; the first generation consists of non-resorbable membranes, while the second generation contains all resorbable membranes [56]. Before Elgali et al. (2017) reviewed this classification and added a new group, Third generation, its membranes rely on naturally derived sources combined with bone grafts and alternative materials to provide structural support to the defect site and to promote the intrinsic regenerative potential of the host tissue [5,15,41].

The following sections discuss the various commercially available periodontal membranes classified as non-resorbable or resorbable materials and are summarized in Table 2.

Table 3 includes a list of the primary biomaterials used in bone tissue engineering, along with their key characteristics.

#### 3.3.1. First-Generation Membranes: Non-Resorbable Guided Membranes

In the 1960s and 1970s, the first generation of barrier membranes were developed with the goal of achieving a sufficient mix of physical qualities that would match those of the replaced tissue while also eliciting a low toxic response in the host [15,41].

In the initial GTR experiments, an occlusive membrane consisting of a bacterial filter made from cellulose acetate (Millipore, Burlington, MA, USA) was utilized. These experiments were conducted by Nyman et al. in 1982 [57]. Due to its toxicity, this form of membrane was not appropriate for clinical applications despite serving its goal. In later trials conducted in the 1990s, membranes of expanded polytetrafluoroethylene (e-PTFE) created specifically for periodontal regeneration were applied (Gore Tex Periodontal Material) [5,15,58].

E-PTFE has a dual-layered structure with pores measuring 5–20 microns in diameter. One side of this membrane is 1 mm thick and has an open microstructure that is 90% porous, preventing epithelial penetration; the other side is 0.15 mm thick and has a porous structure that is 30% thick, allowing space for new bone production [59]. Several investigations have shown that e-PTFE is effective, as described by Liu, J. and Kerns, D.G. [59] However, due to their very porous structural design, they have a high rate of exposure, which is seen as a major disadvantage, in addition to the need for additional surgery to remove them from the location of the newly created tissue.

A high-density d-PTFE membrane with hole sizes of less than 0.3 microns was created to counteract the drawbacks of e-PTFE [60]. In spite of the advantage of non-sticking of tissues to the membrane, which made its removal easy and simple, in addition to its ability to properly regenerate the bones even in exposed cases due to its modified transparency, However, the d-PTFE has limited flexibility, causing it to collapse into the site of the defect [59].

Titanium-reinforced e-PTFE and d-PTFE membranes were produced in order to address the lack of mechanical stiffness that appeared in the initial e-PTFE and d-PTFE membranes [61,62]. However, the requirement for a second surgery to remove the membrane is the most significant disadvantage, similar to other non-resorbable membranes, as well as the rigidity of titanium mesh can create some difficulties during removal due to the need for orthopedic fixation devices such as orthopedic screws. Ti-mesh also appears frequently, which restricts its applications, particularly in aesthetic applications. [63].

#### 3.3.2. Second-Generation Membranes: Resorbable Guided Membranes

Regarding the several applications of GTR and GBR, an absorbable membrane has been proposed as a replacement to the membrane discussed in the previous section in order to minimize its limitations, most notably the requirement for extra surgery to remove the membrane. Based on the origin of the material used to produce the membrane, absorbable membranes are classified into two main groups: natural membranes and synthetic membranes [5,15].

##### Natural Resorbable Membranes

Numerous natural polymers have been shown to be useful in tissue engineering, which include polysaccharides (cellulose, alginate, starch, hyaluronic acid derivatives, chitin/chitosan), and proteins (soy, fibrin gels, collagen, silk) [26,64]. Natural polymers are also strongly coordinated and may include extracellular substances known as ligands that are essential for binding with cell receptors that can support cell adhesion and function. However, on either side, their medicinal use is constrained by their shortage and the complexity of their processing into scaffolds. In addition, they can induce an immune reaction since natural polymers can lead to cells growing at different developmental stages. Moreover, the rate of degradation varies between patients due to the enzymatic processes involved [5,15,65].

Collagen and chitosan appear to be the two main components of most natural membranes, which are naturally derived from many animal sources. Perhaps the most notable one is the use of bovine Achilles tendon (Cytoplast^®^), human skin (Alloderm^®^), or porcine skin (Bio-Gide^®^) to produce tissue-derived membranes based on collagen [66,67].

The presence of collagen in these membranes is a significant biological feature, as it contributes to many biological activities. Besides being biocompatible, biodegradable, and hemostatic, it also helps in attracting the gingival fibroblast and periodontal ligament (PDL) in addition to augmentation of the soft tissue. Using collagen type I, most of the commercially available collagen membranes are produced and developed, as well as a mixture of collagen types I and III [59]. In vivo experiments found that the collagen-dependent membrane showed some drawbacks, such as its modest efficiency, especially during degradation. Moreover, it may cause ethical and religious issues as well as be a cause of disease transmission [30]. Many biophysical characteristics and collagen framework stabilization can be improved by a number of methods that depend mainly on mechanical and chemical cross-linking, such as adding substances such as glutaraldehyde (GA), diphenyl-phosphoryl azide (DPPA), hexamethylene diisocyanate (HMDIC), and formaldehyde (FA), genipin (Gp), in addition to using ultraviolet light and irradiation [15,68,69].

Collagen structural integrity and mechanical properties are affected by the rehydration protocol, i.e., inserting a cross-linking agent that is natural, genipin into the AlloDerm^®^ [68]. Studies have shown that extending the exposure time for genipin (Gp) to 6 h from 30 min significantly improves tensile strength in comparison with controls. Additionally, according to other studies, cross-linking is effective for controlling prolonged biological degradation, decreasing tissue amalgamation, and vascular depression, as well as for decreasing epithelial migration [70]. A biocompatible reaction of the membrane made of silk fibroin by osteoblast was also observed, which could be used for GBR as an alternate barrier membrane [71].

##### Synthetic Resorbable Membranes

Synthetic polymers have several advantages over natural polymers, including the ability to have their properties tuned, an infinite variety of forms, and well-established structures. The support that is provided by synthetic biomaterials can make it possible to restore the structural integrity and functional capacity of diseased or damaged tissues [72]. Synthetic polymers can be modified in terms of their molecular weight, molecular structure, and physical and chemical properties simply unlike polymers derived from natural sources, through the addition of certain functional groups and side chains, synthetic polymers may be self-cross-linked or cross-linked with enzymes or other bioactive molecules [73,74,75].

Synthetic biomaterials have the limitation of lacking cell attachment sites and requiring chemical alterations to improve cell adherence [76]. Physicochemical and mechanical properties of several commercially available synthetic polymers are close to those of biological tissues [77]. The mechanical and physical properties, such as stiffness, Elastic modulus, and degradation rate, are repeatable and predictable throughout a wide spectrum [5,15,76].

The most commonly investigated synthetic degradable materials are poly (-hydroxy esters), which include PCL, PGA, PLA, and their copolymer PLGA, and poly(ethers), which include PEO and PEG, PVA, and PU. These are perhaps the most common examples, however there are now many other synthetic materials being studied [72,76,77]. These polymers all have varying degrees of biodegradability, biocompatibility, and mechanical qualities; nevertheless, there is not a single polymer that possesses all three of these essential properties at the optimal amount [78].

##### PGA-Based Membranes

Polyglycolic acid is organically created through polycondensation of glycolic acid or ring-opening polymerization (ROP) of glycolide. PGA has a very high fusion point at around 226°. PGA can be processed through hydrolysis, and its by-products can be processed by the Krebs cycle and then eliminated. It is generally used as a suture, but it can also be used as a PLA co-polymer [79]. Resolut^®^ is another commercially available product consisting of two layers: a PLGA compact layer that prevents epithelial cell penetration, and a porous network of polyglycolide fibers that promotes tissue integration. Histological studies showed similar effectiveness to non-resorbable membranes and complete resorption 5–6 months after placement [80,81].

Fibers of polyglactin 910, a copolymer of glycolide and L-lactide (9:1 *wt*/*wt*), were used to produce a woven mesh (Vicryl Periodontal Mesh^®^). The polyglactin 910 is inert (no reactions in the surrounding tissue during its adsorption were observed), not antigenic, and preserves its physicomechanical properties during the first 3–4 weeks [82]. Although animal studies indicated a lack of tissue integration and recession formation, clinical evaluation suggested a similar effectiveness as compared to that of other GBR membranes [56,80,81,83].

##### PCL-Based Membranes

Poly-ε-caprolactone is a polymer with some crystal-like properties that melts at approximately 60 °C. It possesses a relatively slow degradation rate, which makes it better suited for long-term applications such as drug delivery systems. A plethora of studies have evaluated this approach and determined that PCL is an effective delivery polymer. Additionally, its physical properties can be modified through the addition of materials such as PGA or PLA. It also possesses applications in osseous scaffolding [84,85,86]. Membranes based on copolymers of lactic acid and e-caprolactone have been produced, showing a lower degradation time as compared to pure PLA membranes. PCL is characterized by higher hydrophobicity and lower water solubility than PLA, PGA and their copolymers. A commercial product, called Vivosorb^®^, consisting of poly(DL-lactide-ecaprolactone), was found to be biocompatible, non-cytotoxic, occlusive and space maintaining [87].

##### PLA-Based Membranes

**Figure 4:**
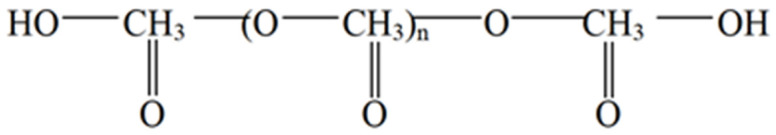
Chemical structure of PLA, where (n) denotes the central repeat unit [modified from [88,89].

Polylactic acid is synthesized similarly to PGA, through ring-opening polymerization of its lactic acid (HOCHCH3COOH) [88]. Its structure can be seen in Figure 4.

PLA is one of the best biopolymers due to its biocompatibility and ease of biological degradation. Because of its properties, it has been used in various biomedical and clinical applications [90,91,92]. PLA exists in three optical isomers, specifically in its L-lactide form as (PLLA) and its D-lactide form as (PDLA). Additionally, it has a hybrid form (PDLLA) [93]. Because of its nature as an amorphous crystal, PDLLA degrades quicker than other forms of PLLA, in less than half a year [94,95].

The Guidor^®^ Matrix Barrier is a bioresorbable membrane, first used for the regeneration of tissues in periodontology, consisting of polylactic acid treated with acetyltributylcitrate to achieve flexibility to guarantee close barrier adaptation to the bone defect. The Guidor^®^ Matrix Barrier has a matrix with two differently perforated layers. The external layer, allowing integration of the overlying gingival flap, presents large pores (rectangular shape) to promote tissue integration and to enable gingival connective tissue to penetrate quickly into the matrix. The inner layer presents small pores (circular shape), able to retard tissue penetration while allowing nutrient permeation. The two layers are separated by many inner spacers, forming an interspace into which tissue can grow. According to the manufacturer, the barrier structure is not affected by the material degradation for at least the first 6 weeks, and a complete resorption takes place after one year due to hydrolysis [56,83].

Atrisorb^®^ membrane is the first liquid product adapted directly at the surgical site: it consists of poly-DL-lactide acid dissolved in N-methyl-2- pyrrolidone. An irregular membrane is produced after polymer exposure to 0.9% saline solution for 4–6 min in a special cassette, in which it is possible to cut it into the desired shape. Membrane thickness is 600–750 µm, and it is positioned into the defect site by applying a moderate pressure. A histological complete resorption was observed 6–12 months after implantation [96]. Clinical studies reported its efficacy in the treatment of periodontal defects [97].

The Epi-Guide^®^ Bioresorbable Barrier Matrix is a porous membrane consisting of D-L polylactic acid with a unique three-layer technology, used as an adjunct to periodontal restorative surgery. The Epi-Guide maintains its structure and functions for 5 months after implantation, with a complete bioresorption after one year [98]. The layer in contact with the gingiva is porous to promote fibroblast infiltration and attachment. On the contrary, the layer in contact with bone defects has a limited porosity that supports fluid uptake, helps adherence to the tooth surface, and inhibits fibroblast movement [98,99].

For successful periodontal tissue regeneration, the materials used must be compatible with living tissue and favorable in terms of mechanical properties. These specifications cannot be fulfilled by conventional single-component polymer materials. As a result, designing and preparing multicomponent polymer structures represents a promising approach for developing multifunctional biomaterials [100].

#### 3.3.3. Third-Generation Membranes

By reviewing the previous absorbable and non-absorbable membranes, interests should arise in developing a new membrane which has a more advanced role as a barrier membrane and has an additional function such as releasing beneficial agents such as bioceramic, antibiotics, growth factors, and adhesion factors into the wound. The substance-releasing membrane should have a proper release time according to the environment of the graft site [9].

##### Resorbable Membranes Based on Polymer Composites

Polymer Blends

Polymer membranes must meet a few key criteria for successful guided bone and tissue regeneration (GBR and GTR, respectively), appropriated mechanical and physical properties, a suitable degradation profile, as well as the necessary strength to provide an effective barrier function and resist decomposition [101]. Due to a variety of requirements, a single polymer fails to meet all critical criteria. For instance, naturally occurring polymers cannot provide the required mechanical strength and suitable degradation profiles, while synthetic polymers are unable to interact with biological tissues. On the other hand, polyester membranes turn rigid and brittle after introduction to phosphate buffered saline or artificial saliva solution [102].

Therefore, the issue of developing membranes with the necessary mechanical properties, the expected rate of decomposition, as well as a structure similar to the natural extracellular matrix (ECM) remains topical [103]. A potential solution is to combine two or more polymers in order to offset their disadvantages and find a mutually reinforcing effect.

Natural Polymer and Synthetic Polymer Blends

Natural polymers are known for their increased biocompatibility and bioactive properties compared to synthetic counterparts. For instance, gelatin shows multiple integrin-binding sites to promote cellular adhesion and differentiation [104,105]. Mixing polymers of natural and synthetic origin should provide opportunities for taking advantage of both of them. For example, a material based on an amalgamation of gelatin with PCL has excellent biocompatibility as well as the essential mechanical, physical, and chemical qualities. Its unique properties allow it to be used in cartilage tissue engineering [106,107], neural tissue engineering [108], as well as GBR and GTR [104,105,109]. That being said, chemical segregation between PCL molecules and gelatin is a factor inhibiting the development of composites with the required characteristics.

It has been found that acetic acid can favorably affect the rate and strength of miscibility between PCL and gelatin. For this reason, it is effective for implementation when homogeneous nanofibers with improved performance are required [110,111]. The biodegradation period of such membranes is also appropriate for tissue regeneration [110].

The PLLA/chitosan multilayer membrane proposed by Ku et al. (2009) has shown excellent potential for utilization in GBR and GTR [112]. The membrane has external chitosan netting that promotes the adhesion of cells from nanoporous PLLA located in the middle layer. This layered structure allows for improved mechanical strength and integrity preservation for up to eight weeks.

Natural Polymers Blends:

Despite its natural origin, the bioactivity and mechanical properties of chitosan are inferior to those of protein polymers. In order to improve its properties, chitosan is often blended with other polymers. Due to the presence of free carboxyl groups in the structure of gelatin, it successfully blends with chitosan and forms a stable hydrogen bond with it. The ability of gelatin/chitosan membranes to maintain cellular adhesion and proliferation is better than that of gelatin and chitosan on their own [113]. Moreover, the enhancement by proanthocyanidin gives the gelatin/chitosan bond greater stability and improves its mechanical properties compared to membranes constructed from gelatin or chitosan and gelatin blend [113]. An example of the successful integration of natural polymers is a three-layer membrane with a chitosan interlayer sandwiched between two collagen membranes featuring 20 wt % HA [114]. Hunter and Ma, (2013) have shown that membranes based on hydroxyapatite/chitosan/gelatin can promote the growth of bone marrow mesenchymal stem cells (hBMSC) whilst improving the pace of osteogenic differentiation [115]. Research data assure that gelatin/chitosan or collagen/chitosan membranes possess adequate mechanical and structural properties to be implemented as a barrier membrane. Therefore, they demonstrate the potential to be used in bone and tissue regeneration.

Synthetic Polymer Blends

PLA, PLGA, PCL, and some other aliphatic polyesters are essential components for the production of fibrous scaffolds required for drug delivery systems and tissue regeneration [116]. At the same time, PLGA is characterized by reduced mechanical strength, which makes it impossible to maintain the scaffolding structure during in vitro and in vivo clinical trials. When PLGA was reinforced with other polymers such as PCL, applied in an equal ratio, the compressive strength of the PCL/PLGA scaffolding was far superior to the strength ensured by PLGA alone [117]. Cytological investigations have demonstrated that penetration of human embryonic kidney 293T cells can be prevented by using PDLLA/PLGA electrospinning devices with an appropriate degradation rate and effective cell occlusion for the purpose of GTR. In addition, implantation of a subcutaneous implant in rats demonstrated that PDLLA/PLGA membranes with a composite ratio of 70/30 and 50/50 are able to double as a physical barrier that stops cellular infiltration for a duration of 13 weeks [103]. These data suggest that PDLLA/PLGA membranes can become an effective barrier membrane for tissue regeneration purposes [103]. Along with this, composite membranes fabricated from PLA/PCL, PLGA/PCL, and other synthetic compounds may be deemed as a promising technology for GBR and GTR [117,118,119].

Floreon™ blend

Floreon is a new sustainable polymer blend created by Floreon-Transforming Packaging Limited in collaboration with the University of Sheffield and certified by the EN13432 standard [120,121]. Based on PLA, Floreon is composed of renewable components, which is likely to improve its mechanical and chemical properties [120,121].

In comparison to pure PLA, Floreon exhibits a remarkable four-fold increase in strength and is less susceptible to cracking and breakage during the manufacturing process and testing phases, as demonstrated by Floreon 3D (2014) and Floreon (2018). The compound has a maximum tensile strength of approximately 1.6 GPa while the elongation at break (fracture strain) is 14% [120,121,122]. Moreover, in comparison to PLA, Floreon exhibits enhanced thermal performance. It has a melting point of 210 °C [120,121,123], a crystallization temperature of 85 °C [123], and a glass transition temperature of 65 °C. Floreon is extruded at temperatures between 170 and 180 °C. However, since its destruction threshold is 250 °C [120,123], technological processes should not exceed 220 °C. In order to prevent moisture absorption, the material is dried at 65–90 °C after crystallization [120]. Floreon may undergo thermoforming, compounding, and injection molding processes in addition to extrusion (including film extrusion).

There are currently eight Floreon variants labelled in the range FL100–FL800 [120,121]. Due to its resilience to ultraviolet radiation, the Floreon blend is more effective than PLA for 3D printing and lithographic printing [120,121].

Although the Floreon was originally designed for the packaging industry, it has recently been investigated as a scaffold for musculoskeletal applications. The conclusion drawn is that the Floreon blend showed great promise for use in bone tissue regeneration [124].

##### Bio-Ceramic/Polymer Composites

The incorporation of polymer composites, bioceramic components, and the structural mimicry of bone extracellular matrix (ECM) can be advantageous for the development of biomaterials that are used for guided bone regeneration (GBR) and guided tissue regeneration (GTR) [9]. Hydroxyapatite (HA) [125], carbonated hydroxyapatite (CHA) [126], bioactive glass (BG) [127], β- tricalcium phosphate (β-TCP) and other bioceramics have been widely used in bone tissue engineering and shown to have excellent biocompatibility and osteoconduction properties [128,129].

The use of bioactive ceramics in GTR and GBR has a positive impact on mineralization and cell activity boost on polymer membranes, which suggests the required osteoconductivity and osteoinductivity [127,130,131,132,133]. On top of that, bioactive compounds are capable of affecting mechanical properties in a beneficial way [134]. While pure PLGA has a tensile strength of 0.49 MPa, the inclusion of 10–30 wt % nanoapatite into a membrane helps lift it to 0.61 MPa [135]. At the same time, the introduction of bioceramics is able to neutralize the acidic derivatives of PLA, chitosan, and other polymers formed due to their decomposition in an alkaline medium [130,136,137]. According to Khan et al. (2008), composite membranes have the ability to effectively and biomimicking preserve the structural and biological functions of damaged dense tissues [138].

Because hydroxyapatite is osteoinductive, it accelerates bone regeneration and allows the bio-ceramics component to connect directly to the regenerated bone, bypassing connective tissue. The composite has found wide application in orthopedic surgery and dentistry dealing with hard tissue restoration [26,139,140]. Inorganic–organic composites that emulate the structure of human bone offer increased toughness inherent in polymeric materials and the compressive strength characteristic of inorganic components. Their beneficial nature makes it possible to create bioactive materials with improved mechanical properties and degradation profiles. Such composites are stable enough since the alkalinity of the inorganic fraction (for example, hydroxyapatite) balances the acidic substances formed during the autocatalytic decomposition of polymers (such as PLA) [141]. Fabricated PCL/nHA nanocomposites possess properties characteristic of HA ceramics and simultaneously provide the qualities of synthetic polymer PCL, namely, osteoconductivity and biocompatibility [26,142]. Studies of poly (lactic acid) (PLA) nanofibers containing hydroxyapatite filler showed that HA contributes to the improvement of the mechanical and thermal features of the nanofibers [143]. In addition, testing of the β-chitin-HA composite membrane made it possible to detect inclusions of apatite on the surface of β-chitin membranes. This finding indicates increased biocompatibility and provides a suitable foundation for successful cell attachment, adhesion, and proliferation [144].

Bioactive glasses (BSs) are osteoconductive and osteoinductive silica biomaterials with a SiO_2_-CaO-P_2_O_5_ structural grid. The introduction of BG stimulates osteogenesis and angiogenesis both in vitro and in vivo [145,146], and also generates high-performance collagen composites in imitation of bone mineralization. In particular, it is involved in the release of Ca, P, and Si and the subsequent deposition of Ca and P as well as amorphous Ca-P crystals on the implant surface. The following chemical dehydration reactions convert these crystals to hydroxycarbonate apatite (HCA) [147]. In a similar way, wollastonite (CaSiO_3_) gives up Si and Ca ions, which induce the acceleration of osteogenic differentiation and cell multiplication. Simultaneously, this can lead to deposits of bone-like apatite on the surface of the implant after it has been introduced to simulated body fluids (SBF) [148,149]. Wollastonite exhibits the capability of increased structural mechanical strength, angiogenesis, and bone regenerative capacity. Despite this, it should be subject to further research to identify the bioactivity, osteogenic capacity, and immunogenicity of polymer composites when implanted in humans. These studies are driving the development of polymer/bioceramic based composites offering the advantages of both components [124].

**Table 2:** List of some commonly used barrier membranes for GTR/GBR therapy.

Resorbability	Barrier Membrane	Composition	Main Characterization	Comments	Refs.
(a) Non-resorbable	Gore-Tex^®^	Expanded polytetrafluoroethylene (e-PTFE).	- Good space maintainer. - Relatively stiff. - Handling.	- Longest clinical experience.	[150,151]
	Cytoplast^®^ TXT-200	High-density polytetrafluoroethylene (d-PTFE).	- Pores with submicron (0.2 μm) size - Density precludes colonization of the host flora and prevents the infection.	- Avoids a second surgery.	[152,153]
	Gore-Tex-TI^®^	Titanium reinforced expanded polytetrafluoroethylene (Ti-e-PTFE).	- Most stable space maintainer, requires no filler material.	Titanium should not be exposed. For recession, ridge augmentation.	[154,155]
(b) Resorbable: natural	Bio-Gide^®^	Collagen derived from porcine skin (types I and III).	- Barrier function At least 6 weeks bioactive.	Usually employed in combination with filler substances	[156,157,158,159]
	BioMend Extend^®^	Collagen type I derived from bovine tendon.	- Resorption: 4–8 weeks - Collagen complexed with formaldehyde	Collagen network extends the resorption time	[160,161]
	AlloDerm^®^	Acellular dermal matrix human skin.	- Resorption: ∼16 weeks. - promoting blood vessel growth, white cell movement, and cell growth.	Ethical concerns and health risks may be associated with the use of human skin.	[9,162]
	Cytoplast ^®^ RTM collagen	Collagen type-I derived from bovine Achilles tendon.	- Resorption: 26–38 weeks. - Multilayered, long-lasting membrane.	Specifically oriented to enhance handling. Possesses good tensile strength.	[9,162]
(c) Resorbable: synthetic	Guidor	Poly-DL-lactid/Poly-L-lactid + acetyltributylcitrate.	- Double-layered membrane. - Outer: large pores. - Inner: finer pores.	- No commercially available.	[56,83,163]
	Resolut	Poly-DL-lactid/Co-glycolid.	- Resorption: 10 weeks. - Functional integrity. - Good space maintainer.	- Good tissue integration-Separate suture material.	[80,81,157]
	Vicryl	Polyglactin 910: Polyglicolid/polylactid 9:1.	- Relatively soft. - Well adaptable. - Resorption: 4–12 weeks.	- Woven membrane. - Four prefabricated shapes.	[82,157,164]
	Atrisorb	Poly-DL-lactide and solvent (N-methyl-2-pyrrolidone).	- Soft Well-adaptable. - Interesting resorptive characteristics.	- Customized membrane fabrication with “Barrier Kit”.	[96,97]
	Epi-Guide	Poly-DL-lactic acid.	- 3-layer technology. - Bioresorption: after 6–12 months.	- Self-supporting, can be used without support from bone grafting materials.	[98,99]
	Vivosorb	Poly (DL-lactide-caprolactone) (PLCL).	- Anti-adhesive barrier. - Maintains its mechanical properties for up to eight weeks.	- Commercially available as a nerve guide.	[87]

**Table 3:** Types of biomaterials used in guided regeneration therapy.

Class	Example	Advantages	Disadvantages	Refs.
Polymers- Natural proteins	Collagen, fibrin, alginate, silk fibroin, hyaluronic acid	Biocompatible Biodegradable without inflammation bioactive	Poor mechanical strength Rapid resorption	[156,160,165,166]
Polymers- Natural Polysaccharides	Chitosan	Biodegradable Biocompatible Has an antibacterial and bioadhesive properties Promote wound healing	Poor mechanical strength Rapid resorption	[167,168,169]
Polymers- Synthetic	Polyglycolic acid (PGA)	Versatile Reproducible Thermoplastic so it can be shaped easily	Inflammatory or immune reaction due to acid release in enzymatic biodegradation Mechanical stability is of limited duration Less biocompatible than natural Not bioactive Rapid resorption Low solubility in organic solvent	[73,170,171,172]
	poly-L-lactide acid (PLLA)	Degrades slower and dissolves easier than PGA Reproducible	The potential to cause immune and foreign-body reactions because it does nor degrade completely The mechanical stability is of limited duration	
	poly-ε-caprolactone (PCL)	Slow degradation rate Reproducible Good workability	Inflammatory or immune reaction Mechanical stability is of limited duration	
*Polymers-Synthetic*	Hydrogel	Modified easily Biocompatible Biodegradable	Contracted Lack stiffness	[73,170,171,172]
*Metal*	Titanium mesh	High mechanical strength and fracture toughness Biocompatible	Corrosion may release toxic particles affecting the biocompatibility and induce an inflammatory reaction Poor stimulation of new bone formation due to the elastic moduli which does not correspond with natural bone	[62,154,173]
*Ceramic*	HA	Biocompatible Osteoconductive Similar to the chemical structure of inorganic phase of bone	Slow biodegradation Difficult to shape due to hardness, fragility, and brittleness	[174,175]
	TCP	Same to above	Rigid and fragile Faster resorption rate	[175,176]
	Bioglass	Biocompatible Osteoconductive Bioactive Promote angiogenesis Enhance cell adhesion and proteins adsorption Easy to control the chemical composition Controlled degradation rate	Brittleness Low resistance to crack due to low strength and fracture toughness	[177,178]
*Composite*	PGA/β-TCP	Better ability for osteogenesis, mineralization and biodegradation than HA	Lack of osteoinductivity	[176]
	Bioglass 45S5 and poly (D, L-lactide) polymer	Improved mechanical properties and resorption rate	Reaction with polymer changes the bioglass surface properties and compromised its bioactivity	[177]
	Poly (b-hydroxybutyrate co-b-hydroxyvalerate) (PHBV) microsphere and poly (L-lactic-coglycolic acid) (PLGA)	Supports drugs and growth factors delivery	Changes in the surface topography and decrease porosity due to dehydration shrinkage	[179]
	Hyaluronic acid-gelatine	Good mechanical propertyBiocompatible High porosity Hydrophilic	Suboptimal cell adhesion due to negative cell-scaffold interaction	[180]
	Nano HA/polymer	Promote better cell adhesion and distribution No significant inflammatory response Biocompatible Improved mechanical properties	Unknown mechanism of cellular proliferation and differentiation	[50]

##### Multiphasic Scaffolds of Periodontal Tissues Regeneration

A multiphasic scaffold is defined by the differences in its architecture (porosity, pore organization, etc.) and its chemical composition, which usually mimics to some degree the structure or cellular and biochemical composition of the native tissue. Multiphasic scaffolds are designed to impart biomimetic functionality to tissue-engineered bone and soft tissue grafts have been recognized for some time as having the potential to facilitate clinical translation in the field of orthopedic tissue engineering, and more recently in the field of periodontal tissue regeneration [181].

In recent years, guided tissue regeneration and guided bone regeneration (GTR and GBR) approaches have been widely used to manage periodontitis. These membranes have separate functions on each side. The occlusive periodontal membrane acts as a barrier to inhibit the ingrowth of epithelial and undesirable tissues into the defective area during periodontal wound healing, whereas the opposite side promotes regeneration of periodontal tissues [181,182]. GTR/GBR membranes must have certain features, particularly those utilized in large-area repair, such as mechanical stability, osteoconductivity, and a balance between membrane degradation and tissue regeneration, all of which are required for the membranes to function [181]. In a number of studies, bilayer GTR/GBR membranes have been utilized as a treatment for periodontal diseases; here are a few examples from the last few years.

The Yoshimoto group has recently developed bilayer membranes based on PLGA or PCL [183,184]. These membranes consisted of a solid layer and a porous layer that, respectively, served as a barrier and provided cell support. By changing the freeze-drying temperature, they were able to control the thickness of each layer. These membranes were found to be more functional than monolayer membranes, with evidence suggesting that their porous structure aided in the osteogenic differentiation and proliferation of mesenchymal stem cells. In vivo studies also demonstrated that the PLGA bilayer membrane promoted bone regeneration with significantly increased bone formation compared to that with a monolayer membrane [184].

Requicha et al. (2016), in a related method, created a biphasic scaffold made of a porous fibrous PCL/starch scaffold for enabling bone ingrowth and an occlusive membrane developed using the same matter [185]. In this technique also, the occlusive membrane was devised to sustain periodontal ligament regeneration by inhibiting epithelial and gingival tissue invasion of the periodontal defect, hence carefully choosing osteoblast and periodontal fibroblast ingrowth as per the GTR law. After performing in vitro analyses, Requicha et al. (2014) and Requicha et al. (2016) discovered a high potential for osteogenesis, which is a key aspect of periodontal regeneration [185,186].

Park et al. (2010) suggested an approach that involves computer-assisted design and manufacturing (CAD/CAM), using two dissimilar sacrificial instruments to 3D print a mold with the negative imprint of the scaffold design. This method directly uses additive manufacturing technology to create a biphasic scaffold consisting of bone and ligament compartments [187]. Later, polymer solutions specific to each compartment (polyglycolic acid and polycaprolactone for bone and ligament compartments, respectively) were tossed into these molds. Consequently, the solvent evaporated before getting rid of the sacrificial material. The resultant porous scaffold had defined dimensions and shape and a definite internal pore architecture. In the process of developing the two compartments independently, they were consequently gathered by utilizing a thin PCL film, hence developing into a biphasic scaffold [187]. The researchers used fibrin to deliver BMP-7-transfected human gingival fibroblasts and human periodontal fibroblasts into the bone and periodontal ligament compartments, correspondingly. The usefulness of the cellularized biphasic scaffold was monitored by means of a murine ectopic model while a human dentin block was placed in the periodontal ligament compartment. This process showed that the presence of periodontal cells to a high degree enabled the attachment of a freshly developed ligament onto the dentine slice together with the sedimentation of cementum-like tissue 6 weeks before implantation [187].

Focusing on guided bone regeneration, which is the focal point of this project, Zhang et al. (2019) have recently studied the most commonly utilized GBR membrane, known as Bio-Gide, which is among the most commonly used commercial biodegradable membranes, and has a wide range of advantages [181]. Bio-Gide possesses a bilayer makeup in which one of the sides is structured to be compact and soft to inhibit epithelium and connective tissue interference on the other side of bone defects, and the opposite side is permeable and coarse to enable the bond of osteoblasts next to the bone defect.

The aforementioned experts described a unique form of multifunctional GBR membrane with similar design characteristics as those of the Bio-Gide membrane but including extra roles that the Bio-Gide membrane cannot accomplish. The unique GBR membrane is made up of a compact nacre-like coating and a permeable membrane. The function of the nacre-like layer is to give great mechanical properties and also to inhibit non-osteoblast interference. Conversely, the porous layer has been designed with the aim of necessitating osteoblast adhesion. For a number of reasons, they asserted that their multifunctional nanocomposite membrane was better than the other GBR membranes. These reasons include biocompatibility combination with the facial surface, high mechanical performance, sufficient rate of degradation, and efficacious bacteriostasis. For these reasons, this type of nanocomposite membrane qualifies to be considered as a perfect bioactive GBR membrane for medical use [181,188].

By combining the electrospinning technique with emulsion templating, a bilayer barrier membrane (BM) made of a biodegradable synthetic polymer, PCL, was effectively developed by [189]. Some of the qualities exhibited by the resultant BM included the absence of delamination, a qualitatively resistant structure to twisting and elongation, and simplicity in handling. The electrospun layer of the BM has been proven to possess the ability to act as a barrier, offering protection to the bone defect against soft tissue interference. On the other hand, the interconnected PCL polyHIPE layer has exhibited pivotal characteristics to be the bone-enhancing layer, supplying crucial needs including boosting collagen and mineral deposition and enhancing cellular infiltration and cell compatibility [189].

### 3.4. Scaffold Manufacturing Technologies

Scaffolding manufacturing is a highly nuanced and evolving field, encompassing a diverse array of techniques tailored to various applications and material requirements. The process of creating porous scaffolds is far from uniform, with methodologies varying significantly in complexity, cost, and final product quality. This section delves into a selection of prevalent manufacturing technologies, each catering to specific needs and producing results unique in structure and functionality. Distinct from existing studies, such as the one referenced in citation [190,191,192,193,194,195,196,197,198,199,200], this review aims to not only present an overview of established techniques but also emphasize recent advancements, novel applications, and critical evaluations. From the rapid yet modest-quality processes to intricate and time-intensive methods that yield superior structures, we provide an insightful and contemporary analysis, with a particular focus on how these methods contribute to various applications, including bone regeneration. This comprehensive examination serves as a valuable resource for researchers and practitioners seeking an up-to-date understanding of scaffolding manufacturing technologies.

#### 3.4.1. Solid Free-Form Fabrication Technique

SFFT is a manufacturing technique also recognized as rapid prototyping (RP), which also refers to a type of fabrication process called additive manufacturing [191]. In which components are printed by depositing one cross-section layer over the other layer and assembled using a three-dimensional computer-aided design (CAD) model [190]. Three-dimensional scaffolds with complex geometries and dimensionally accurate structures can be manufactured using data obtained from medical scans and then adjusted to meet the needs of each individual patient [190,191].

This process is accomplished through several phases. The first phase is based on creating a computer-aided design (CAD) model, which is then sent to a file that can be manipulated with a stereolithography apparatus. Automatically, the STL file is divided into horizontal layers throughout the pre-production phase. Then, printing continues in this layered process. The final structure needs to be hardened and its surface treated before being used [191]. Through the use of sophisticated scanning techniques such as magnetic resonance imaging (MRI) or computer tomography (CT) [191]. These highly detailed 3D images can then be used to make the creation of precise [26], integrated scaffolds [192] and significantly reproducible [191]. This is particularly useful when making highly porous structures at approximately 90% or more of the total volume of the scaffold [192].

Scaffolds with sophisticated and controlled macro-and microporous structures can be provided by SFFT, potentially both within the same structure [26]. Table 4 compares the various SFFT types, which have been evaluated by different research groups [191,192,193,194]. This list includes their inherent advantages and disadvantages. SFFT is a modern development. It helps in creating solutions rather quickly, but not all types of SFFT can be used for scaffold manufacturing [191,192,193,194].

#### 3.4.2. Three-Dimensional Bioprinting Technique

Three-dimensional (3D) bioprinting is a sophisticated and intricate method of additive manufacturing that meticulously incorporates a range of biological materials to generate structures which resemble and function such as living tissues. This technique is known for its scalability, meaning it can be adjusted to create complex structures that meet individual patients’ specific needs in terms of size and complexity. Furthermore, the technology enables the precise distribution of cellular components-including but not limited to growth factors, proteins, cells, and drug particles. These favorable conditions have spearheaded advancements in several medically and clinically relevant applications such as drug testing, high-throughput assays, tissue engineering, tissue regeneration, and cancer research [195].

One of the most challenging tasks in 3D bioprinting is the creation of blood vessels and organs. The complexity of this task arises from the need to integrate diverse cell types, the constraint of limited structural support, and the requirements of a concomitant capillary network, which is a typical characteristic of functional organs. Despite the considerable technological advances, these multifaceted requirements make the printing of such structures a significant challenge [196].

Nonetheless, relentless research efforts have led to some notable advancements. Researchers have successfully printed rudimentary structures, such as blood vessels, skin, and cartilage that does not require a blood supply. These achievements mark promising milestones in the field of 3D bioprinting [196]. Attempts have also been made to print bone tissue, specifically bone that comprises its natural constituents such as nerve and muscle tissue. Despite these endeavors, the structures produced are yet to match the functional superiority of their naturally occurring counterparts. Therefore, it is evident that continued research and development are needed to overcome the existing challenges in 3D bioprinting [197].

#### 3.4.3. Gas-Foaming Technique

**Figure 5:**
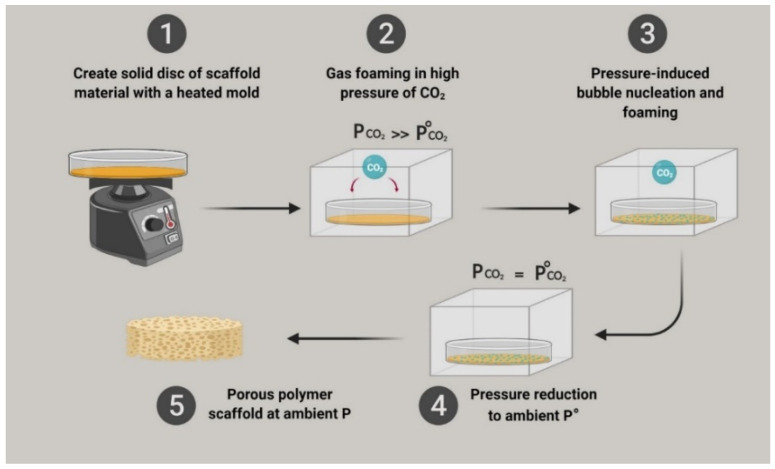
Schematic illustration of the gas-foaming technique, [source: [200], redesigned with copyright permission from Elsevier license number: 4724150945567, dated 8 December 2019].

With this technique, there is no longer a need to use solvents that are normally present in the previously mentioned methods. This method creates a porous network through the dispersion of gas bubbles that, when the material is hardened, act as pores, as illustrated in Figure 5. A heated mold is used to heat the polymer material, which is usually made of polylactic-co-glycolic acid, which is then molded by compressing it to make rigid discs. After this, these molded structures are pumped with high pressure (5.5 MPa) CO_2_ for 3 days at 25 °C. Afterward, gas pressure is reduced to atmospheric levels and, therefore, gas solubility is reduced. This process makes CO_2_ gas create inner clumps, which then create the pores needed for the proper function of the implant. This method allows for the total number of porosities to reach up to 93% and sizes of approximately 100 mm. It is not trivial, however, to control pore size and interconnectivity with this technique [198,199].

#### 3.4.4. Thermally Induced Phase Separation Technique

**Figure 6:**
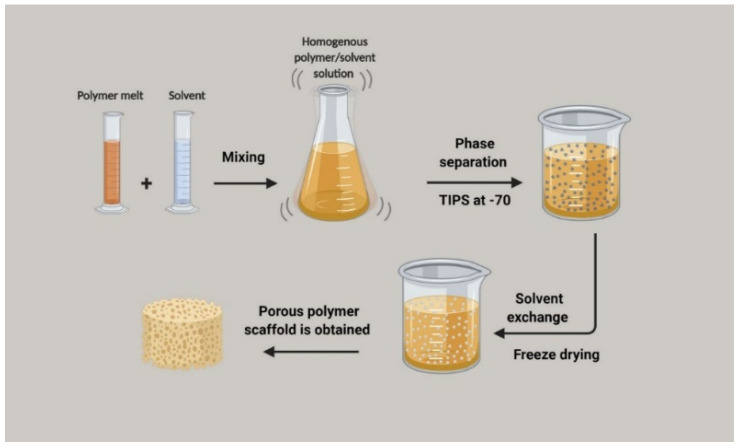
Schematic representation of the porous scaffold fabrication process with the Thermally Induced Phase Separation (TIPS), [source: [200], redesigned with copyright permission from Elsevier license number: 4724150945567, dated 8 December 2019].

A procedure that allows for the fabrication of highly porous anisotropic scaffolds is called Thermally Induced Phase Separation (TIPS). These polymer scaffolds can be controlled with ease but have a low ability to be applied to affected tissues such as ligaments, muscles, nerves, intestines, and osseous structures [201]. Depending on the concentration of polymer used, certain characteristics will change, such as mechanical properties, pore shape, biological activity, and the rate of resorption. Furthermore, these properties will change depending on the volume of the phase separation [190]. A polymer phase fraction can be achieved by dissolving a polymer at a high degree of temperature in a certain solvent, then cooling the homogenous polymer/solvent solution to obtain a polymer porous scaffold. After this process is completed, a microporous scaffold can be obtained immediately after the solvent has evaporated, as schematically shown in Figure 6 [201].

#### 3.4.5. Emulsion Freeze-Drying Technique

**Figure 7:**
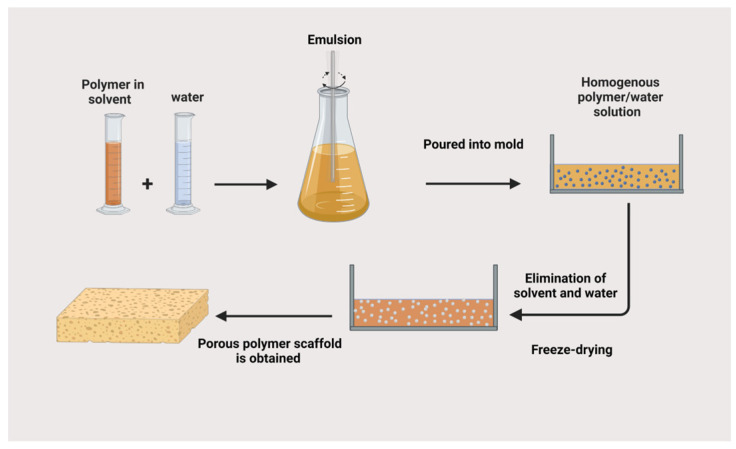
Schematic illustration of the freeze-drying process, [source: [200], redesigned with copyright permission from Elsevier license number: 4724150945567, dated 8 December 2019].

This technique is based on the phase fraction through the use of different physical properties of the fiber by emulsifying the solution and then drying it at a very low temperature [202], and producing a scaffold that has abundant pores, as illustrated in Figure 7.

The first step in this process is the creation of the emulsion by homogenizing a polymer in a carbon-based solvent and water. This emulsion must be quickly frozen and the formed phases (solvent and water) are then eliminated by freeze-drying the sample. The resulting polymer scaffolds will have pores of between 20 and 200 µm [190]. This method could be combined with the third method that was mentioned, as well as adding crystal-forming polar compounds such as sucrose or NaCl, in order to further increase porosity. Once the sample has been dried, these particles can be cleared with the use of water [167].

#### 3.4.6. Solvent Casting and Particulate Leaching Technique

**Figure 8:**
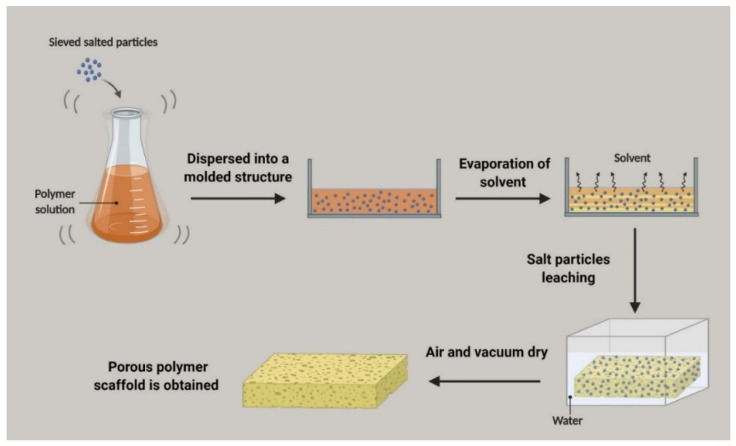
Schematic illustration of the solvent casting and particulate leaching technique, [source: [200], redesigned with copyright permission from Elsevier license number: 4724150945567, dated 8 December 2019].

Another common method for making scaffolds is solvent casing. This process starts with the deconstruction of a polymer in a carbon-based solvent, as schematically shown in Figure 8. The aforementioned method uses “porogens”, a group of chemical compounds that can be distributed into a structure during the manufacturing process and then taken away through the use of water, leaving behind a porous structure. These porogens can create a coupled polymer-porogen structure when added to the overall solution. As soon as the polymer reaches its final form and starts hardening, and the original solvent evaporates away, water is then used to dissolve porogens, which is often a high polarity compound, such as NaCl. Although it is hard to control the final inner structure of the scaffold since it is difficult to predict and control where the porogen particles will be distributed and then dissolved, a three-dimensional porous polymer scaffold was obtained [199,203].

#### 3.4.7. Spin-Coating Technique

The spin coating technique is a widely used method for depositing thin films of materials onto a flat substrate. In this technique, a liquid solution containing the scaffold material is dispensed onto the substrate, which is then rotated at high speeds (typically in the range of 1000 to 4000 rpm). The centrifugal force generated by the spinning substrate causes the solution to spread out evenly over the substrate, forming a thin film. The speed and duration of the spinning process can be controlled to achieve a desired thickness and uniformity of the film.

**Figure 9:**
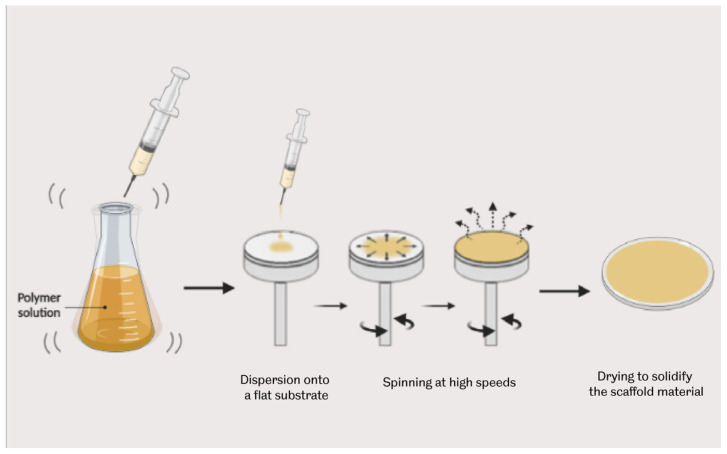
Schematic illustration of the spin coating technique, illustrating the steps involved in fabricating a scaffold.

In Figure 9, a schematic outline of the spin coating process is shown. The figure depicts the substrate, the spin coater, and the solution being applied to the substrate. As the substrate is spun, the centrifugal force pulls the solution towards the edges, creating a thin and uniform film on the substrate.

The advantages of the spin coating technique include its simplicity and cost-effectiveness, as well as its ability to produce films with precise control over their thickness and uniformity. This technique can also be easily combined with other techniques to create complex multilayered structures, such as for periodontal tissue regeneration.

Limitations of the preceding manufacturing techniques

In practice, the techniques used to manufacture scaffolds are divided into solid free-form fabrication and conventional methods. Each of them produces various scaffolds with distinctive characteristics [204]. Even though SFFT provides a plethora of potential opportunities for tissue engineering and possesses undeniable advantages, there are some inherent drawbacks that must be considered. Firstly, each method uses a very specific fabrication material. SLS uses a fine powder, whereas the use of thermoplastics is more efficient for FDM. Even when the selected material is appropriate, if it is difficult to prepare, it can make the whole process much more challenging. Secondly, the fact that a material can be successfully printed does not guarantee its proper function, since successful scaffolds also require constructs to maintain their integrity throughout their layers. The material must be able to support itself after its fabrication, maintaining its integrity layer by layer. Thirdly, in the case of the printing of biological tissues, novel material solidification techniques that are used to preserve the fabricated scaffold integrity should be developed [205]. Lastly, when using materials that are cell-loaded, the flexibility of print parameters such as shear stress or temperature is restricted. This happens since cell environments are ever changing, and doing so would be deleterious for cell survival [206].

While conventional techniques of scaffolding fabrication include the construction of porous polymer structures such as substrates for cell adhesion, it is difficult to obtain complex structures with tunable microscale and macroscale using conventional methods [207]. In addition, some of these methods are manual based. Therefore, they are labor intensive and difficult to reproduce. Another limitation is the need for organic solvents and porogens, which are cytotoxic and their residues may cause inflammatory responses [208]. Benefits and limitations of conventional and Solid free-form manufacturing techniques are discussed and summarized in Table 5 [188,209].

#### 3.4.8. Electrospinning for Bone Regeneration

Over the last ten years, electrospinning technology has become one of the most interesting methods for creating scaffolds used for tissue engineering. The creation of these nanofiber scaffolds has become the focus of research for many investigators due to their many unique properties, especially those which are clinically relevant. In particular, this method is used in order to manufacture nanofibers used in different applications in dentistry such as tooth restoration, repair of oral mouth tissue, preventing tooth decay, and restoring other dental and periodontal tissues, such as the repair of dentin, endodontium, oral mucosa, periodontal tissue, as well as alveolar bone regeneration [210].

A type of material that has received a lot of attention recently has been biodegradable polymers, especially in biomedical areas such as bio-prosthetics, tissue engineering, and the application of drug delivery systems. Aliphatic polyesters are one of the most significant types of synthetic biodegradable polymers, owing in particular to their advantageous characteristics of biocompatibility and biodegradability. The main allure of these polymers (polyesters) is their biological compatibility and their ability to be degraded within the organ [211].

Electrostatic spinning, most commonly known as electrospinning, has been a focal point of research for the last 20 years due to the various potential uses of the created microfiber in both nanotechnologies and nanoscience [212]. Notable characteristics, such as high permeability, large surface-to-volume ratio, and excellent pore interconnection, have made electrospun microfiber ideal for normal cell functions, such as nutrient and cell transportation [213,214].

**Figure 10:**
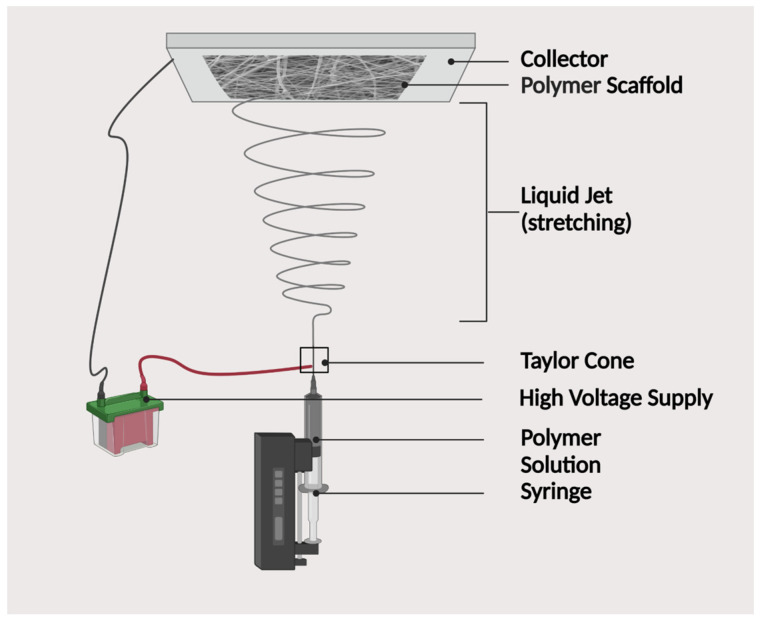
Example of electrospinning apparatus, source: [200], redesigned with copyright permission from Elsevier license number: 4724150945567, dated 8 December 2019.

Additionally, the nanofibrous scaffolds manufactured through this method can provide excellent extracellular conditions, such as coupling, migration, and cell proliferation, especially for those cells in charge of hard tissue repair. Along with the simplicity of setup and cost efficiency, the opportunity to create microfiber with a large variety of physical and chemical properties is its own merit. This machine consists of a syringe needle, a grounded collector (metal plate), a high-voltage electrical source, and a syringe pump (Figure 10).

The electrical source must carry around 10 to 30 kilovolts and is applied to solutions that are ejected via the syringe needle. When the electrical charge reaches the starting point, the surface tension of the charged solution begins to change, causing a deformation of the solution droplet into a conical droplet known as the Taylor cone. While the electrical force overcomes the surface tension of the charged solution, thin charged jets are ejected from the tip of the metallic needle in a nearly straight line towards the electrically inverse electrode. As the material is being extruded, the solvent is being evaporated away, resulting in the construction of continuous dry polymer fiber, which leads to the formation of a non-woven surface of the obtained fiber. The grounded collector surface is generally placed approximately 20 cm from the syringe’s tip [215].

## 4. Future Directions and Conclusions

The dynamic landscape of guided tissue and bone regeneration is primarily driven by the interplay of biomaterials and scaffold manufacturing technologies, offering a diverse array of solutions for periodontal therapeutic interventions. As we move forward, several trends and directions can be envisioned that are expected to shape the future trajectory of this field.

One such direction is the exploration of more advanced, next-generation membranes, which could potentially surpass the benefits offered by the current third-generation membranes. The development of these membranes could focus on enhanced biocompatibility, optimized resorption rates, and improved mechanical strength. For instance, research on Floreon™ blend and other innovative polymer combinations that offer superior mechanical strength and enhanced bioresorbability may provide novel solutions for effective tissue regeneration [124]. A focus on personalized treatment approaches utilizing patient-specific 3D bioprinting technologies may also prove beneficial [195].

Furthermore, the integration of growth factors or other bioactive molecules within these membranes to promote bone and tissue regeneration is a promising area of exploration. With recent advances in drug delivery technologies, the controlled and localized delivery of these factors could significantly enhance therapeutic outcomes [49,170]. A notable recent development is the use of Zn-based biodegradable materials in biomedical applications. With their potential to serve as next-generation orthopedic implants, Zn-based materials may offer significant advantages over conventional alternatives, such as reducing the need for revision surgeries and minimizing biocompatibility issues [216]. These materials have demonstrated a significant role in bone metabolism and new cell growth, and they show medium degradation without the release of excessive hydrogen. The addition of alloying elements such as Mg, Zr, Mn, Ca, and Li into pure Zn has been found to enhance the mechanical properties of Zn alloys, making them a promising material for future guided tissue and bone regeneration applications [216]. Additionally, there is an increasing interest in nanotechnologies and their application in bone regeneration. The fabrication of nanocomposite scaffolds can potentially improve the cellular response and lead to better mimicking of the natural extracellular matrix. Such improvements could foster bone formation and promote better integration with host tissue [50,181].

In the realm of scaffold manufacturing technologies, there is considerable scope for enhancing precision and reducing production times, perhaps through the advancement of computer-aided design and manufacturing (CAD/CAM) techniques. Hybrid fabrication methods that combine the advantages of different techniques can also be explored, such as combining electrospinning with 3D printing, to create complex scaffold architectures with improved mechanical properties and porosity [187].

The future direction of guided tissue and bone regeneration is also increasingly being influenced by the emerging field of tissue engineering, specifically the use of stem cells. Combining biomaterials with stem cell therapy could lead to unprecedented advancements in periodontal tissue regeneration, offering high-potential treatment strategies that need to be explored extensively [217].

In conclusion, guided tissue and bone regeneration represents an exciting and rapidly advancing field with vast potential for further innovation and improvement. The progression in this field will rely heavily on a comprehensive understanding of biomaterials and their interactions with biological systems, as well as the continued refinement of manufacturing technologies. Embracing an interdisciplinary approach, encompassing materials science, biology, engineering, and clinical dentistry will be essential for the successful evolution of periodontal treatments.

## Figures and Tables

**Table 1:** Bone graft classification by material source. adapted from [20,21].

Type	Source		Benefit	Risk
Autograft	Patient		Osteogenic, osteoinductive and osteoconductive No immunological rejection living cells and matrices	Morbidity at donor sites Amount of bone volume is limited. Rapid absorption
Allograft	Another human	Demineralized freeze-dried bone allograft (DFDBA)	Osteoinductive and osteoconductive	Potential of infection and immunological rejection
		Freeze-dried bone allograft (FDBA)	Osteoinductive and osteoconductive	Potential of infection and immunological rejection
Xenograft	Other species (Mostly bovine)		Osteoconductive	Potential of infection and immunological rejection Slow resorption or non-resorbable
Alloplast	Synthetic	Sintered hydroxyapatite (HA)	Osteoconductive	Slow resorption or non-resorbable
		β-tricalcium phosphate (β-TCP)	Osteoconductive	Rapid resorption
		Natural products (coral, chitosan, etc.)	Osteoconductive, low immunological rejection	Slow resorption or non-resorbable

**Table 4:** Different types of SFFT with their advantages and disadvantages.

Techniques	Materials	Advantages	Disadvantages	Refs.
Stereolithography (SL)	PEG, PEGDA, PPF, PCL, PDLLA	High accuracy, complex 3D structure including agents and cells, easy removal of photopolymer by heating	Photo-polymerization of materials, photocurable materials, expensive materials and equipment	[191,192,193,194]
Fused deposition modelling (FDM)	Thermoplastic polymers and their composites (PVA, ABSP400)	High porosity, complete pore interconnectivity, possibility of controlling porosity and size of pores, macro shape control, good compressive strength, solvent-free	High processing temperature, limited material range, inconsistency in pores	
Selective laser sintering (SLS)	Polymer ceramics (PCL, HAp, TCP)	Complex structure, possibility of controlling porosity and size of pores independently, wide range of powder materials, solvent-free, any secondary binder system	High processing temperature, using only thermally stable polymers, limited to small pore size	
3D printing (3D-P)	Ceramics, polymers, metals	Easy process, high porosity, complete pore interconnectivity, possibility of controlling porosity and size of pores independently, macro shape control, wide range of materials	Use of toxic organic solvent, lack of mechanical strength, limited to small pore size	

**Table 5:** Comparison of different scaffold fabrication techniques: advantages and disadvantages, adapted from [188,209].

Manufacturing Method	Benefits	Potential Limitations
Gas foaming	- Eliminates use of chemical solvents.	- High pressures involved prohibits inclusion of cells and bioactive molecules directly into scaffolds - Temperature labile materials may be denatured during compression molding step - Difficult to control pore sizes and ensure interconnectivity
Emulsification freeze-drying	- Does not require use of solid porogen.	- Requires use of organic solvents - Small pore size and - Porosity often irregular - Long processing time
Phase separation	- Eliminates leaching step of porogen - Can be combined with other techniques easily.	- Small pore sizes limit use - Use of organic solvents inhibits use of bioactive molecules or cells during scaffold fabrication
3D Printing - SLA - Inkjet - SLS - Laser-assisted - FDM - Microvalve - Micro-extrusion	- Complex 3D shapes with high resolution, controlled pore size and morphology and controlled internal structures can be fabricated. Improved capacity to incorporate vascular structures into constructs. - Depending on technique used, cells may be included in high concentration directly in scaffold materials.	- Some techniques are limited by printable materials - Set up costs can be expensive for machinery
Solvent casting/ particulate leaching	- Relatively simple technique that allows creation of scaffolds with regular porosity, controlled composition and pore size.	- Use of organic solvents precludes cells and biomolecules being included directly in scaffolds - Can be difficult to control pore shape and interconnectivity - Limited thickness of structures and mechanical properties achievable
Spin-coating	- Simple and cost-effective method. - Precise control over the thickness and uniformity of the film.	- Requires optimization of parameters such as the spinning speed and duration for each specific material and substrate combination
Electrospinning	- Essential technique for developing nanofibrous scaffolds for the TE. - Homogeneous mixture made of fiber with high tensile strength. - Simple instrument. - Continuous process. - Cost effective compared to other existing methods. - Scalable. - Ability to fabricate fiber diameters few nm to several microns.	- Used solvents can be toxic - Jet instability - Packaging, shipping, handling

## Data Availability

All data analyzed during this study are included in this published article.

## Abbreviations

%	Percentage
°C	Degree Celsius
BMP	Bone morphogenic protein
Ca	Calcium
CAD/CAM	Computer-aided design and manufacturing
CaSiO_3_	Calcium silicate
CO	Carbon monoxide
conc	Concentration
CT	Computer tomography
DFDBA	Demineralized freeze-dried bone allograft
DPPA	Diphenyl-phosphoryl azide
EMD	Enamel matrix derivative
FA	Formaldehyde
FDBA	Freeze-dried bone allograft
FDM	Fused deposition modelling
GA	Glutaraldehyde
GBR	Guided bone regeneration
gm or g	Gram
Gp	Genipin
GTR	Guided tissue regeneration
HA	Hydroxyapatite
HMDIC	Hexamethylene diisocyanate
IGF	Insulin-like growth factor
mg	Milligram
mm	Millimeter
MPa	Megapascal
MRI	Magnetic resonance imaging
MSCs	Mesenchymal stem cells
Mw	Molecular weight
Na	Sodium
NOFs	Normal oral fibroblast cells
-OH	Hydroxyl
PCL	Poly-ε-caprolactone
PDGF	Platelet derived growth factor
PDL	Periodontal ligaments
PGA	Polyglycolic acid
PLA	Polylactic acid
PLGA	Poly (lactic-co-glycolic acid)
PO_4_	Phosphate
PTFE	Polytetrafluoroethylene
ROP	Ring-opening polymerization
SBF	Simulated body fluid
SD	Standard deviation
SFFT	Solid free-form fabrication technique
SLS	Selective laser sintering
TCP	Tri calcium phosphate
TGF-beta	Transforming growth factor-beta
TIPS	Thermally induced phase separation
βTCP	Beta tricalcium phosphate

## References

[1] Huck O., Stutz C., Gegout P.-Y., Özçelik H., Benkirane-Jessel N., Petit C., Batool F. (2022). Nanomedicine and Periodontal Regenerative Treatment. Dent. Clin. N. Am..

[2] Kinane D.F., Stathopoulou P.G., Papapanou P.N. (2017). Periodontal diseases. Nat. Rev. Dis. Prim..

[3] Liu Y., Guo L., Li X., Liu S., Du J., Xu J., Hu J., Liu Y. (2022). Challenges and tissue engineering strategies of periodontal-guided tissue regeneration. Tissue Eng. Part C Methods.

[4] Dimitriou R., Mataliotakis G.I., Calori G.M., Giannoudis P.V. (2012). The role of barrier membranes for guided bone regeneration and restoration of large bone defects: Current experimental and clinical evidence. BMC Med..

[5] Elgali I., Omar O., Dahlin C., Thomsen P. (2017). Guided bone regeneration: Materials and biological mechanisms revisited. Eur. J. Oral Sci..

[6] Esposito M., Grusovin M.G., Felice P., Karatzopoulos G., Worthington H.V., Coulthard P. (2009). Interventions for replacing missing teeth: Horizontal and vertical bone augmentation techniques for dental implant treatment. Cochrane Database Syst. Rev..

[7] Chen F.-M., Zhang J., Zhang M., An Y., Chen F., Wu Z.-F. (2010). A review on endogenous regenerative technology in periodontal regenerative medicine. Biomaterials.

[8] Yuan H., Fernandes H., Habibovic P., De Boer J., Barradas A.M.C., De Ruiter A., Walsh W.R., Van Blitterswijk C.A., De Bruijn J.D. (2010). Osteoinductive ceramics as a synthetic alternative to autologous bone grafting. Proc. Natl. Acad. Sci. USA.

[9] Bottino M.C., Thomas V., Schmidt G., Vohra Y.K., Chu T.-M.G., Kowolik M.J., Janowski G.M. (2012). Recent advances in the development of GTR/GBR membranes for periodontal regeneration—A materials perspective. Dent. Mater..

[10] Dahlin C., Linde A., Gottlow J., Nyman S. (1988). Healing of bone defects by guided tissue regeneration. Plast. Reconstr. Surg..

[11] Taba M., Jin Q., Sugai J.V., Giannobile W.V. (2005). Current concepts in periodontal bioengineering. Orthod. Craniofac. Res..

[12] Sculean A., Nikolidakis D., Schwarz F. (2008). Regeneration of periodontal tissues: Combinations of barrier membranes and grafting materials–biological foundation and preclinical evidence: A systematic review. J. Clin. Periodontol..

[13] Liang Y., Luan X., Liu X. (2020). Recent advances in periodontal regeneration: A biomaterial perspective. Bioact. Mater..

[14] Needleman I., Tucker R., Giedrys-Leeper E., Worthington H. (2002). A systematic review of guided tissue regeneration for periodontal infrabony defects. J. Periodont. Res..

[15] Lee H.-S., Byun S.-H., Cho S.-W., Yang B.-E. (2019). Past, present, and future of regeneration therapy in oral and periodontal tissue: A review. Appl. Sci..

[16] Giannobile W.V. (2014). Commentary: Treatment of periodontitis: Destroyed periodontal tissues can be regenerated under certain conditions. J. Periodontol..

[17] Melcher A.H. (1976). On the repair potential of periodontal tissues. J. Periodontol..

[18] Dhruvakumar D., Arun Kumar K.V., Ayilavarapu S. (2019). Hand Book on Bone Regeneration: Materials, Techniques and Procedures: From Research to Clinical Practice.

[19] Nyman S., Lang N.P., Buser D., Brägger U. (1990). Bone regeneration adjacent to titanium dental implants using guided tissue regeneration: A report of two cases. Int. J. Oral Maxillofac. Implants.

[20] McAllister B.S., Haghighat K. (2007). Bone augmentation techniques. J. Periodontol..

[21] Zhao R., Yang R., Cooper P.R., Khurshid Z., Shavandi A., Ratnayake J. (2021). Bone grafts and substitutes in dentistry: A review of current trends and developments. Molecules.

[22] Mellonig J.T. (1992). Autogenous and allogeneic bone grafts in periodontal therapy. Crit. Rev. Oral Biol. Med..

[23] Miron R.J., Zhang Y.F. (2012). Osteoinduction: A review of old concepts with new standards. J. Dent. Res..

[24] Urist M.R. (1965). Bone: Formation by autoinduction. Science.

[25] Richardson C.R., Mellonig J.T., Brunsvold M.A., McDonnell H.T., Cochran D.L. (1999). Clinical evaluation of Bio-Oss^®^: A bovine-derived xenograft for the treatment of periodontal osseous defects in humans. J. Clin. Periodontol..

[26] Rezwan K., Chen Q.Z., Blaker J.J., Boccaccini A.R. (2006). Biodegradable and bioactive porous polymer/inorganic composite scaffolds for bone tissue engineering. Biomaterials.

[27] Schmidlin P.R., Nicholls F., Kruse A., Zwahlen R.A., Weber F.E. (2013). Evaluation of moldable, in situ hardening calcium phosphate bone graft substitutes. Clin. Oral Implants Res..

[28] Damien E., Revell P.A. (2004). Coralline hydroxyapatite bone graft substitute: A review of experimental studies and biomedical applications. J. Appl. Biomater. Biomech..

[29] Koo K.-T., Polimeni G., Pringle G.A., Agelan A., Safadi F.F., Wikesjö U.M.E. (2008). Histopathological observations of a polylactic acid-based device intended for guided bone/tissue regeneration. Clin. Implant. Dent. Relat. Res..

[30] Behring J., Junker R., Walboomers X.F., Chessnut B., Jansen J.A. (2008). Toward guided tissue and bone regeneration: Morphology, attachment, proliferation, and migration of cells cultured on collagen barrier membranes. A systematic review. Odontology.

[31] Agrawal C.M., Parr J.E., Lin S.T. (2000). Synthetic Bioabsorbable Polymers for Implants.

[32] Gottlow J., Nyman S., Lindhe J., Karring T., Wennström J. (1986). New attachment formation in the human periodontium by guided tissue regeneration Case reports. J. Clin. Periodontol..

[33] Gottlow J., Nyman S., Karring T., Lindhe J. (1984). New attachment formation as the result of controlled tissue regeneration. J. Clin. Periodontol..

[34] Needleman I., Worthington H.V., Giedrys-Leeper E., Tucker R. (2006). Guided tissue regeneration for periodontal infra-bony defects. Cochrane Database Syst. Rev..

[35] Cortellini P., Prato G.P., Tonetti M.S. (1996). Periodontal regeneration of human intrabony defects with bioresorbable membranes. A controlled clinical trial. J. Periodontol..

[36] Murphy K.G., Gunsolley J.C. (2003). Guided tissue regeneration for the treatment of periodontal intrabony and furcation defects. A systematic review. Ann. Periodontol..

[37] Buser D., Dula K., Belser U., Hirt H.-P., Berthold H. (1993). Localized ridge augmentation using guided bone regeneration. I. Surgical procedure in the maxilla. Int. J. Periodontics Restor. Dent..

[38] Slavkin H.C., Bartold P.M. (2006). Challenges and potential in tissue engineering. Periodontology 2000.

[39] Wikesjö U.M.E., Crigger M., Nilvéus R., Selvig K.A. (1991). Early healing events at the dentin-connective tissue interface. Light and transmission electron microscopy observations. J. Periodontol..

[40] Wikesjö U.M.E., Nilvéus R.E., Selvig K.A. (1992). Significance of early healing events on periodontal repair: A review. J. Periodontol..

[41] Nuñez J., Vignoletti F., Caffesse R.G., Sanz M. (2019). Cellular therapy in periodontal regeneration. Periodontology 2000.

[42] Pitaru S., McCulloch C.A.G., Narayanan S.A. (1994). Cellular origins and differentiation control mechanisms during periodontal development and wound healing. J. Periodontal Res..

[43] McClellan S.J., Franses E.I. (2005). Adsorption of bovine serum albumin at solid/aqueous interfaces. Colloids Surfaces A Physicochem. Eng. Asp..

[44] Schenk R.K., Buser D., Hardwick W.R., Dahlin C. (1994). Healing pattern of bone regeneration in membrane-protected defects: A histologic study in the canine mandible. Int. J. Oral Maxillofac. Implants.

[45] Lang H., Schiiler N., Arnhold S., Nolden R., Mertens T. (1995). Formation of differentiated tissues in vivo by periodontal cell populations cultured in vitro. J. Dent. Res..

[46] Lin W., McCulloch C.A.G., Cho M. (1994). Differentiation of periodontal ligament fibroblasts into osteoblasts during socket healing after tooth extraction in the rat. Anat. Rec..

[47] Boyko G.A., Melcher A.H., Brunette D.M. (1981). Formation of new periodontalligament by periodontal ligament cells implanted in vivo after culture in vitro: A preliminary study of transplanted roots in the dog. J. Periodontal Res..

[48] Wang H.-L., MacNeil R.L. (1998). Guided tissue regeneration. Absorbable barriers. Dent. Clin. N. Am..

[49] Gao Y., Wang S., Shi B., Wang Y., Chen Y., Wang X., Lee E.-S., Jiang H.-B. (2022). Advances in modification methods based on biodegradable membranes in guided bone/tissue regeneration: A review. Polymers.

[50] Basile M.A., d’Ayala G.G., Malinconico M., Laurienzo P., Coudane J., Nottelet B., Della Ragione F., Oliva A. (2015). Functionalized PCL/HA nanocomposites as microporous membranes for bone regeneration. Mater. Sci. Eng. C.

[51] Karring T. (2000). Regenerative periodontal therapy. J. Int. Acad. Periodontol..

[52] Sheikh Z., Khan A.S., Roohpour N., Glogauer M., u Rehman I. (2016). Protein adsorption capability on polyurethane and modified-polyurethane membrane for periodontal guided tissue regeneration applications. Mater. Sci. Eng. C.

[53] Kim K.-H., Jeong L., Park H.-N., Shin S.-Y., Park W.-H., Lee S.-C., Kim T.-I., Park Y.-J., Seol Y.-J., Lee Y.-M. (2005). Biological efficacy of silk fibroin nanofiber membranes for guided bone regeneration. J. Biotechnol..

[54] Laycock B., Nikolić M., Colwell J.M., Gauthier E., Halley P., Bottle S., George G. (2017). Lifetime prediction of biodegradable polymers. Prog. Polym. Sci..

[55] Brydone A.S., Meek D., Maclaine S. (2010). Bone grafting, orthopaedic biomaterials, and the clinical need for bone engineering. Proc. Inst. Mech. Eng. Part H J. Eng. Med..

[56] Gottlow J. (1993). Guided tissue regeneration using bioresorbable and non-resorbable devices: Initial healing and long-term results. J. Periodontol..

[57] Nyman S., Gottlow J., Karring T., Lindhe J. (1982). The regenerative potential of the periodontal ligament: An experimental study in the monkey. J. Clin. Periodontol..

[58] Aaboe M., Pinholt E.M., Hjorting-Hansen E. (1995). Healing of experimentally created defects: A review. Br. J. Oral Maxillofac. Surg..

[59] Liu J., Kerns D.G. (2014). Suppl 1: Mechanisms of guided bone regeneration: A review. Open Dent. J..

[60] Madhuri S.V. (2016). Membranes for Periodontal Regeneration. Int. J. Pharm. Sci. Invent..

[61] Canullo L., Malagnino V.A. (2008). Vertical Ridge Augmentation Around Implants by e-PTFE Titanium-Reinforced Membrane and Bovine Bone Matrix: A 24-to 54-Month Study of 10 Consecutive Cases. Int. J. Oral Maxillofac. Implants.

[62] Fontana F., Santoro F., Maiorana C., Iezzi G., Piattelli A., Simion M. (2008). Clinical and Histologic Evaluation of Allogeneic Bone Matrix Versus Autogenous Bone Chips Associated with Titanium-Reinforced e-PTFE Membrane for Vertical Ridge Augmentation: A Prospective Pilot Study. Int. J. Oral Maxillofac. Implants.

[63] Sam G., Pillai B.R.M. (2014). Evolution of barrier membranes in periodontal regeneration-“are the third generation membranes really here?. J. Clin. Diagn. Res..

[64] Peter M., Ganesh N., Selvamurugan N., Nair S.V., Furuike T., Tamura H., Jayakumar R. (2010). Preparation and characterization of chitosan–gelatin/nanohydroxyapatite composite scaffolds for tissue engineering applications. Carbohydr. Polym..

[65] Cheung H.-Y., Lau K.-T., Lu T.-P., Hui D. (2007). A critical review on polymer-based bio-engineered materials for scaffold development. Compos. Part B Eng..

[66] Felipe M.E.M.C., Andrade P.F., Grisi M.F.M., Souza S.L.S., Taba M., Palioto D.B., Novaes A.B. (2007). Comparison of two surgical procedures for use of the acellular dermal matrix graft in the treatment of gingival recessions: A randomized controlled clinical study. J. Periodontol..

[67] Bottino M.C., Jose M.V., Thomas V., Dean D.R., Janowski G.M. (2009). Freeze-dried acellular dermal matrix graft: Effects of rehydration on physical, chemical, and mechanical properties. Dent. Mater..

[68] Bottino M.C., Thomas V., Jose M.V., Dean D.R., Janowski G.M. (2010). Acellular dermal matrix graft: Synergistic effect of rehydration and natural crosslinking on mechanical properties. J. Biomed. Mater. Res. Part B Appl. Biomater..

[69] Sundararaghavan H.G., Monteiro G.A., Lapin N.A., Chabal Y.J., Miksan J.R., Shreiber D.I. (2008). Genipin-induced changes in collagen gels: Correlation of mechanical properties to fluorescence. J. Biomed. Mater. Res. Part A Off. J. Soc. Biomater. Jpn. Soc. Biomater. Aust. Soc. Biomater. Korean Soc. Biomater..

[70] Schwarz F., Rothamel D., Herten M., Sager M., Becker J. (2006). Angiogenesis pattern of native and cross-linked collagen membranes: An immunohistochemical study in the rat. Clin. Oral Implants Res..

[71] Yoo C.-K., Jeon J.-Y., Kim Y.-J., Kim S.-G., Hwang K.-G. (2016). Cell attachment and proliferation of osteoblast-like MG63 cells on silk fibroin membrane for guided bone regeneration. Maxillofac. Plast. Reconstr. Surg..

[72] Reddy M.S.B., Ponnamma D., Choudhary R., Sadasivuni K.K. (2021). A comparative review of natural and synthetic biopolymer composite scaffolds. Polymers.

[73] Place E.S., George J.H., Williams C.K., Stevens M.M. (2009). Synthetic polymer scaffolds for tissue engineering. Chem. Soc. Rev..

[74] Zhu J. (2010). Bioactive modification of poly (ethylene glycol) hydrogels for tissue engineering. Biomaterials.

[75] Geckil H., Xu F., Zhang X., Moon S., Demirci U. (2010). Engineering hydrogels as extracellular matrix mimics. Nanomedicine.

[76] Kluge J.A., Mauck R.L. (2011). Synthetic/biopolymer nanofibrous composites as dynamic tissue engineering scaffolds. Biomedical Applications of Polymeric Nanofibers.

[77] Cascone M.G., Barbani N., Giusti C.C.P., Ciardelli G., Lazzeri L. (2001). Bioartificial polymeric materials based on polysaccharides. J. Biomater. Sci. Polym. Ed..

[78] Ciardelli G., Chiono V., Vozzi G., Pracella M., Ahluwalia A., Barbani N., Cristallini C., Giusti P. (2005). Blends of poly-(ε-caprolactone) and polysaccharides in tissue engineering applications. Biomacromolecules.

[79] Liu H., Slamovich E.B., Webster T.J. (2005). Increased osteoblast functions on nanophase titania dispersed in poly-lactic-co-glycolic acid composites. Nanotechnology.

[80] Pontoriero R., Wennström J., Lindhe J. (1999). The use of barrier membranes and enamel matrix proteins in the treatment of angular bone defects. A prospective controlled clinical study. J. Clin. Periodontol..

[81] Donos N., Kostopoulos L., Karring T. (2002). Alveolar ridge augmentation using a resorbable copolymer membrane and autogenous bone grafts: An experimental study in the rat. Clin. Oral Implants Res..

[82] De Sanctis M., Zucchelli G. (1996). Guided tissue regeneration with a resorbable barrier membrane (Vicryl) for the management of buccal recession: A case report. Int. J. Periodontics Restor. Dent..

[83] Araujo M.G., Berglundh T., Lindhe J. (1998). GTR treatment of degree III furcation defects with 2 different resorbable barriers an experimental study in dogs. J. Clin. Periodontol..

[84] Marra K.G., Szem J.W., Kumta P.N., DiMilla P.A., Weiss L.E. (1999). In vitro analysis of biodegradable polymer blend/hydroxyapatite composites for bone tissue engineering. J. Biomed. Mater. Res. Off. J. Soc. Biomater. Jpn. Soc. Biomater. Aust. Soc. Biomater. Korean Soc. Biomater..

[85] Li W., Danielson K.G., Alexander P.G., Tuan R.S. (2003). Biological response of chondrocytes cultured in three-dimensional nanofibrous poly (ϵ-caprolactone) scaffolds. J. Biomed. Mater. Res. Part A Off. J. Soc. Biomater. Jpn. Soc. Biomater. Aust. Soc. Biomater. Korean Soc. Biomater..

[86] Sinha V.R., Bansal K., Kaushik R., Kumria R., Trehan A. (2004). Poly-ϵ-caprolactone microspheres and nanospheres: An overview. Int. J. Pharm..

[87] Meek M.F., Jansen K., Steendam R., Oeveren W.V., van Wachem P.B., van Luyn M.J.A. (2004). In vitro degradation and biocompatibility of poly (dl-lactide-ϵ-caprolactone) nerve guides. J. Biomed. Mater. Res. Part A An Off. J. Soc. Biomater. Japanese Soc. Biomater. Aust. Soc. Biomater. Korean Soc. Biomater..

[88] Conn R.E., Kolstad J.J., Borzelleca J.F., Dixler D.S., Filer L.J., LaDu B.N., Pariza M.W. (1995). Safety assessment of polylactide (PLA) for use as a food-contact polymer. Food Chem. Toxicol..

[89] Garlotta D. (2001). A literature review of poly (lactic acid). J. Polym. Environ..

[90] Rokkanen P.U., Böstman O., Hirvensalo E., Mäkelä E.A., Partio E.K., Pätiälä H., Vainionpää S., Vihtonen K., Törmälä P. (2000). Bioabsorbable fixation in orthopaedic surgery and traumatology. Biomaterials.

[91] Georgiou G., Mathieu L., Pioletti D.P., Bourban P., Månson J., Knowles J.C., Nazhat S.N. (2007). Polylactic acid–phosphate glass composite foams as scaffolds for bone tissue engineering. J. Biomed. Mater. Res. Part B Appl. Biomater. Off. J. Soc. Biomater. Jpn. Soc. Biomater. Aust. Soc. Biomater. Korean Soc. Biomater..

[92] Gupta B., Revagade N., Hilborn J. (2007). Poly (lactic acid) fiber: An overview. Prog. Polym. Sci..

[93] Kim K., Yu M., Zong X., Chiu J., Fang D., Seo Y.-S., Hsiao B.S., Chu B., Hadjiargyrou M. (2003). Control of degradation rate and hydrophilicity in electrospun non-woven poly (D, L-lactide) nanofiber scaffolds for biomedical applications. Biomaterials.

[94] Merolli A., Gabbi C., Cacchioli A., Ragionieri L., Caruso L., Giannotta L., Leali P.T. (2001). Bone response to polymers based on poly-lactic acid and having different degradation times. J. Mater. Sci. Mater. Med..

[95] Chen Y.-W., Umeda M., Nagasawa T., Takeuchi Y., Huang Y., Inoue Y., Iwai T., Izumi Y., Ishikawa I. (2008). Periodontitis may increase the risk of peripheral arterial disease. Eur. J. Vasc. Endovasc. Surg..

[96] Hou L., Yan J., Tsai A.Y., Lao C., Lin S., Liu C. (2004). Polymer-assisted regeneration therapy with Atrisorb^®^ barriers in human periodontal intrabony defects. J. Clin. Periodontol..

[97] Camargo P.M., Lekovic V., Weinlaender M., Vasilic N., Madzarevic M., Kenney E.B. (2002). Platelet-rich plasma and bovine porous bone mineral combined with guided tissue regeneration in the treatment of intrabony defects in humans. J. Periodontal Res..

[98] Takata T., Miyauchi M., Wang H. (2001). Migration of osteoblastic cells on various guided bone regeneration membranes. Clin. Oral Implants Res..

[99] Bilir A., Aybar B., Tanrikulu S.H., Issever H., Tuna S. (2007). Biocompatibility of different barrier membranes in cultures of human CRL 11372 osteoblast-like cells: An immunohistochemical study. Clin. Oral Implants Res..

[100] Christenson E.M., Anseth K.S., van den Beucken J.J.J.P., Chan C.K., Ercan B., Jansen J.A., Laurencin C.T., Li W., Murugan R., Nair L.S. (2007). Nanobiomaterial applications in orthopedics. J. Orthop. Res..

[101] Zupancic S., Kocbek P., Baumgartner S., Kristl J. (2015). Contribution of nanotechnology to improved treatment of periodontal disease. Curr. Pharm. Des..

[102] Owen G.R., Jackson J.K., Chehroudi B., Brunette D.M., Burt H.M. (2010). An in vitro study of plasticized poly (lactic-co-glycolic acid) films as possible guided tissue regeneration membranes: Material properties and drug release kinetics. J. Biomed. Mater. Res. Part A.

[103] Zhang E., Zhu C., Yang J., Sun H., Zhang X., Li S., Wang Y., Sun L., Yao F. (2016). Electrospun PDLLA/PLGA composite membranes for potential application in guided tissue regeneration. Mater. Sci. Eng. C.

[104] Shi R., Xue J., He M., Chen D., Zhang L., Tian W. (2014). Structure, physical properties, biocompatibility and in vitro/vivo degradation behavior of anti-infective polycaprolactone-based electrospun membranes for guided tissue/bone regeneration. Polym. Degrad. Stab..

[105] Xue J., He M., Liang Y., Crawford A., Coates P., Chen D., Shi R., Zhang L. (2014). Fabrication and evaluation of electrospun PCL–gelatin micro-/nanofiber membranes for anti-infective GTR implants. J. Mater. Chem. B.

[106] Xue J., Feng B., Zheng R., Lu Y., Zhou G., Liu W., Cao Y., Zhang Y., Zhang W.J. (2013). Engineering ear-shaped cartilage using electrospun fibrous membranes of gelatin/polycaprolactone. Biomaterials.

[107] Zheng R., Duan H., Xue J., Liu Y., Feng B., Zhao S., Zhu Y., Liu Y., He A., Zhang W. (2014). The influence of Gelatin/PCL ratio and 3-D construct shape of electrospun membranes on cartilage regeneration. Biomaterials.

[108] Gupta D., Venugopal J., Prabhakaran M.P., Dev V.R.G., Low S., Choon A.T., Ramakrishna S. (2009). Aligned and random nanofibrous substrate for the in vitro culture of Schwann cells for neural tissue engineering. Acta Biomater..

[109] Ji W., Yang F., Ma J., Bouma M.J., Boerman O.C., Chen Z., van den Beucken J.J.J.P., Jansen J.A. (2013). Incorporation of stromal cell-derived factor-1α in PCL/gelatin electrospun membranes for guided bone regeneration. Biomaterials.

[110] Xue J., He M., Liu H., Niu Y., Crawford A., Coates P.D., Chen D., Shi R., Zhang L. (2014). Drug loaded homogeneous electrospun PCL/gelatin hybrid nanofiber structures for anti-infective tissue regeneration membranes. Biomaterials.

[111] Feng B., Tu H., Yuan H., Peng H., Zhang Y. (2012). Acetic-acid-mediated miscibility toward electrospinning homogeneous composite nanofibers of GT/PCL. Biomacromolecules.

[112] Ku Y., Shim I.K., Lee J.Y., Park Y.J., Rhee S., Nam S.H., Park J.B., Chung C.P., Lee S.J. (2009). Chitosan/poly (l-lactic acid) multilayered membrane for guided tissue regeneration. J. Biomed. Mater. Res. Part A Off. J. Soc. Biomater. Jpn. Soc. Biomater. Aust. Soc. Biomater. Korean Soc. Biomater..

[113] Kim S., Nimni M.E., Yang Z., Han B. (2005). Chitosan/gelatin–based films crosslinked by proanthocyanidin. J. Biomed. Mater. Res. Part B Appl. Biomater. Off. J. Soc. Biomater. Jpn. Soc. Biomater. Aust. Soc. Biomater. Korean Soc. Biomater..

[114] Teng S., Lee E., Wang P., Shin D., Kim H. (2008). Three-layered membranes of collagen/hydroxyapatite and chitosan for guided bone regeneration. J. Biomed. Mater. Res. Part B Appl. Biomater. Off. J. Soc. Biomater. Jpn. Soc. Biomater. Aust. Soc. Biomater. Korean Soc. Biomater..

[115] Hunter K.T., Ma T. (2013). In vitro evaluation of hydroxyapatite–chitosan–gelatin composite membrane in guided tissue regeneration. J. Biomed. Mater. Res. Part A.

[116] Jiao Y.-P., Cui F.-Z. (2007). Surface modification of polyester biomaterials for tissue engineering. Biomed. Mater..

[117] Kim J.Y., Yoon J.J., Park E.K., Kim D.S., Kim S.-Y., Cho D.-W. (2009). Cell adhesion and proliferation evaluation of SFF-based biodegradable scaffolds fabricated using a multi-head deposition system. Biofabrication.

[118] Vaquette C., Cooper-White J. (2013). A simple method for fabricating 3-D multilayered composite scaffolds. Acta Biomater..

[119] Xu H., Cui W., Chang J. (2013). Fabrication of patterned PDLLA/PCL composite scaffold by electrospinning. J. Appl. Polym. Sci..

[120] Floreon 3D. “Floreon 3D.” 2014. https://www.floreon.com/floreon3d.

[121] Floreon. Ltd (2018). Floreon-Transforming Packag. https://www.floreon.com.

[122] (2018). Plastics—Determination of Tensile Properties—Part 3: Test Conditions for Films and Sheets.

[123] Lee C.H., Sapuan S.M., Hassan M.R. (2018). Thermal analysis of kenaf fiber reinforced floreon biocomposites with magnesium hydroxide flame retardant filler. Polym. Compos..

[124] Ramos-Rodriguez D.H., Pashneh-Tala S., Bains A.K., Moorehead R.D., Kassos N., Kelly A.L., Paterson T.E., Orozco-Diaz C.A., Gill A.A., Ortega Asencio I. (2022). Demonstrating the Potential of Using Bio-Based Sustainable Polyester Blends for Bone Tissue Engineering Applications. Bioengineering.

[125] Bottino M.C., Thomas V., Janowski G.M. (2011). A novel spatially designed and functionally graded electrospun membrane for periodontal regeneration. Acta Biomater..

[126] Liao S., Wang W., Uo M., Ohkawa S., Akasaka T., Tamura K., Cui F., Watari F. (2005). A three-layered nano-carbonated hydroxyapatite/collagen/PLGA composite membrane for guided tissue regeneration. Biomaterials.

[127] Rowe M.J., Kamocki K., Pankajakshan D., Li D., Bruzzaniti A., Thomas V., Blanchard S.B., Bottino M.C. (2016). Dimensionally stable and bioactive membrane for guided bone regeneration: An in vitro study. J. Biomed. Mater. Res. Part B Appl. Biomater..

[128] Shim J.-H., Yoon M.-C., Jeong C.-M., Jang J., Jeong S.-I., Cho D.-W., Huh J.-B. (2014). Efficacy of rhBMP-2 loaded PCL/PLGA/β-TCP guided bone regeneration membrane fabricated by 3D printing technology for reconstruction of calvaria defects in rabbit. Biomed. Mater..

[129] Shim J.-H., Huh J.-B., Park J.Y., Jeon Y.-C., Kang S.S., Kim J.Y., Rhie J.-W., Cho D.-W. (2013). Fabrication of blended polycaprolactone/poly (lactic-co-glycolic acid)/β-tricalcium phosphate thin membrane using solid freeform fabrication technology for guided bone regeneration. Tissue Eng. Part A.

[130] Mota J., Yu N., Caridade S.G., Luz G.M., Gomes M.E., Reis R.L., Jansen J.A., Walboomers X.F., Mano J.F. (2012). Chitosan/bioactive glass nanoparticle composite membranes for periodontal regeneration. Acta Biomater..

[131] Yang F., Both S.K., Yang X., Walboomers X.F., Jansen J.A. (2009). Development of an electrospun nano-apatite/PCL composite membrane for GTR/GBR application. Acta Biomater..

[132] Leal A.I., Caridade S.G., Ma J., Yu N., Gomes M.E., Reis R.L., Jansen J.A., Walboomers X.F., Mano J.F. (2013). Asymmetric PDLLA membranes containing Bioglass^®^ for guided tissue regeneration: Characterization and in vitro biological behavior. Dent. Mater..

[133] Zhao X., Wu Y., Du Y., Chen X., Lei B., Xue Y., Ma P.X. (2015). A highly bioactive and biodegradable poly (glycerol sebacate)–silica glass hybrid elastomer with tailored mechanical properties for bone tissue regeneration. J. Mater. Chem. B.

[134] Li W., Ding Y., Yu S., Yao Q., Boccaccini A.R. (2015). Multifunctional chitosan-45S5 bioactive glass-poly (3-hydroxybutyrate-co-3-hydroxyvalerate) microsphere composite membranes for guided tissue/bone regeneration. ACS Appl. Mater. Interfaces.

[135] Jamuna-Thevi K., Saarani N.N., Kadir M.R.A., Hermawan H. (2014). Triple-layered PLGA/nanoapatite/lauric acid graded composite membrane for periodontal guided bone regeneration. Mater. Sci. Eng. C.

[136] Peter M., Binulal N.S., Nair S.V., Selvamurugan N., Tamura H., Jayakumar R. (2010). Novel biodegradable chitosan–gelatin/nano-bioactive glass ceramic composite scaffolds for alveolar bone tissue engineering. Chem. Eng. J..

[137] Qasim S.B., Delaine-Smith R.M., Fey T., Rawlinson A., Rehman I.U. (2015). Freeze gelated porous membranes for periodontal tissue regeneration. Acta Biomater..

[138] Khan A.S., Ahmed Z., Edirisinghe M.J., Wong F.S.L., Rehman I.U. (2008). Preparation and characterization of a novel bioactive restorative composite based on covalently coupled polyurethane–nanohydroxyapatite fibres. Acta Biomater..

[139] Bianco A., Cacciotti I., Lombardi M., Montanaro L., Gusmano G. (2007). Thermal stability and sintering behaviour of hydroxyapatite nanopowders. J. Therm. Anal. Calorim..

[140] Porter A.E., Patel N., Skepper J.N., Best S.M., Bonfield W. (2004). Effect of sintered silicate-substituted hydroxyapatite on remodelling processes at the bone–implant interface. Biomaterials.

[141] Ferraz M.P., Monteiro F.J., Manuel C.M. (2004). Hydroxyapatite nanoparticles: A review of preparation methodologies. J. Appl. Biomater. Biomech..

[142] Bianco A., Di Federico E., Moscatelli I., Camaioni A., Armentano I., Campagnolo L., Dottori M., Kenny J.M., Siracusa G., Gusmano G. (2009). Electrospun poly (ε-caprolactone)/Ca-deficient hydroxyapatite nanohybrids: Microstructure, mechanical properties and cell response by murine embryonic stem cells. Mater Sci. Eng. C.

[143] Sonseca A., Peponi L., Sahuquillo O., Kenny J.M., Giménez E. (2012). Electrospinning of biodegradable polylactide/hydroxyapatite nanofibers: Study on the morphology, crystallinity structure and thermal stability. Polym. Degrad. Stab..

[144] Madhumathi K., Binulal N.S., Nagahama H., Tamura H., Shalumon K.T., Selvamurugan N., Nair S.V., Jayakumar R. (2009). Preparation and characterization of novel β-chitin–hydroxyapatite composite membranes for tissue engineering applications. Int. J. Biol. Macromol..

[145] Gorustovich A.A., Roether J.A., Boccaccini A.R. (2010). Effect of bioactive glasses on angiogenesis: A review of in vitro and in vivo evidences. Tissue Eng. Part B Rev..

[146] Xynos I.D., Edgar A.J., Buttery L.D.K., Hench L.L., Polak J.M. (2001). Gene-expression profiling of human osteoblasts following treatment with the ionic products of Bioglass^®^ 45S5 dissolution. J. Biomed. Mater. Res. Off. J. Soc. Biomater. Jpn. Soc. Biomater. Aust. Soc. Biomater. Korean Soc. Biomater..

[147] Izquierdo-Barba I., Arcos D., Sakamoto Y., Terasaki O., López-Noriega A., Vallet-Regí M. (2008). High-performance mesoporous bioceramics mimicking bone mineralization. Chem. Mater..

[148] Li X., Chang J. (2005). Preparation and characterization of bioactive collagen/wollastonite composite scaffolds. J. Mater. Sci. Mater. Med..

[149] Wang X., Zhou Y., Xia L., Zhao C., Chen L., Yi D., Chang J., Huang L., Zheng X., Zhu H. (2015). Fabrication of nano-structured calcium silicate coatings with enhanced stability, bioactivity and osteogenic and angiogenic activity. Colloids Surf. B Biointerfaces.

[150] Villar C.C., Cochran D.L. (2010). Regeneration of periodontal tissues: Guided tissue regeneration. Dent. Clin..

[151] Becker W., Becker B.E., Handelsman M., Ochsenbein C., Albrektsson T. (1991). Guided tissue regeneration for implants placed into extraction sockets: A study in dogs. J. Periodontol..

[152] Bartee B.K. (1995). The use of high-density polytetrafluoroethylene membrane to treat osseous defects: Clinical reports. Implant Dent..

[153] Barber H.D., Lignelli J., Smith B.M., Bartee B.K. (2007). Using a dense PTFE membrane without primary closure to achieve bone and tissue regeneration. J. Oral Maxillofac. Surg..

[154] Jovanovic S.A., Nevins M. (1995). Bone formation utilizing titanium-reinforced barrier membranes. Int. J. Periodontics Restor. Dent..

[155] Lindfors L.T., Tervonen E.A.T., Sándor G.K.B., Ylikontiola L.P. (2010). Guided bone regeneration using a titanium-reinforced ePTFE membrane and particulate autogenous bone: The effect of smoking and membrane exposure. Oral Surg. Oral Med. Oral Pathol. Oral Radiol. Endodontology.

[156] Taguchi Y., Amizuka N., Nakadate M., Ohnishi H., Fujii N., Oda K., Nomura S., Maeda T. (2005). A histological evaluation for guided bone regeneration induced by a collagenous membrane. Biomaterials.

[157] Zhao S., Pinholt E.M., Madsen J.E., Donath K. (2000). Histological evaluation of different biodegradable and non-biodegradable membranes implanted subcutaneously in rats. J. Cranio-Maxillofac. Surg..

[158] Zwahlen R.A., Cheung L.K., Zheng L., Chow R.L.K., Li T., Schuknecht B., Grätz K.W., Weber F.E. (2009). Comparison of two resorbable membrane systems in bone regeneration after removal of wisdom teeth: A randomized-controlled clinical pilot study. Clin. Oral Implants Res..

[159] Gielkens P.F.M., Schortinghuis J., De Jong J.R., Raghoebar G.M., Stegenga B., Bos R.R.M. (2008). Vivosorb^®^, Bio-Gide^®^, and Gore-Tex^®^ as barrier membranes in rat mandibular defects: An evaluation by microradiography and micro-CT. Clin. Oral Implants Res..

[160] Sela M.N., Kohavi D., Krausz E., Steinberg D., Rosen G. (2003). Enzymatic degradation of collagen-guided tissue regeneration membranes by periodontal bacteria. Clin. Oral Implants Res..

[161] Maksoud M.A. (2001). Immediate implants in fresh posterior extraction sockets: Report of two cases. J. Oral Implantol..

[162] Sheikh Z., Abdallah M.N., Hamdan N., Javaid M.A., Khurshid Z., Matilinna K. (2014). Barrier membranes for tissue regeneration and bone augmentation techniques in dentistry. Handbook of Oral Biomaterials.

[163] Miller N., Penaud J., Foliguet B., Membre H., Ambrosini P., Plombas M. (1996). Resorption rates of 2 commercially available bioresorbable membranes: A histomorphometric study in a rabbit model. J. Clin. Periodontol..

[164] Thoma D.S., Halg G., Dard M.M., Seibl R., Hammerle C.H.F., Jung R.E. (2009). Evaluation of a new biodegradable membrane to prevent gingival ingrowth into mandibular bone defects in minipigs. Clin. Oral Implants Res..

[165] Thomas V., Dean D.R., Jose M.V., Mathew B., Chowdhury S., Vohra Y.K. (2007). Nanostructured biocomposite scaffolds based on collagen coelectrospun with nanohydroxyapatite. Biomacromolecules.

[166] Wikesjö U.M.E., Qahash M., Huang Y., Xiropaidis A., Polimeni G., Susin C. (2009). Bone morphogenetic proteins for periodontal and alveolar indications; biological observations–clinical implications. Orthod. Craniofac. Res..

[167] Alizadeh M., Abbasi F., Khoshfetrat A.B., Ghaleh H. (2013). Microstructure and characteristic properties of gelatin/chitosan scaffold prepared by a combined freeze-drying/leaching method. Mater. Sci. Eng. C.

[168] Pighinelli L., Kucharska M. (2013). Chitosan–hydroxyapatite composites. Carbohydr. Polym..

[169] Thein-Han W.W., Kitiyanant Y. (2007). Chitosan scaffolds for in vitro buffalo embryonic stem-like cell culture: An approach to tissue engineering. J. Biomed. Mater. Res. Part B Appl. Biomater. Off. J. Soc. Biomater. Jpn. Soc. Biomater. Aust. Soc. Biomater. Korean Soc. Biomater..

[170] Nair L.S., Laurencin C.T. (2007). Biodegradable polymers as biomaterials. Prog. Polym. Sci..

[171] Agrawal C.M., McKinney J.S., Huang D., Athanasiou K.A. (2000). The use of the vibrating particle technique to fabricate highly porous and permeable biodegradable scaffolds. Synthetic Bioabsorbable Polymers for Implants.

[172] Bhattarai D., Aguilar L., Park C., Kim C. (2018). A review on properties of natural and synthetic based electrospun fibrous materials for bone tissue engineering. Membranes.

[173] Kedem S., Schmidt J., Paz Y., Cohen Y. (2005). Composite polymer nanofibers with carbon nanotubes and titanium dioxide particles. Langmuir.

[174] Sun F., Zhou H., Lee J. (2011). Various preparation methods of highly porous hydroxyapatite/polymer nanoscale biocomposites for bone regeneration. Acta Biomater..

[175] Sulaiman S.B., Keong T.K., Cheng C.H., Saim A.B., Idrus R.B.H. (2013). Tricalcium phosphate/hydroxyapatite (TCP-HA) bone scaffold as potential candidate for the formation of tissue engineered bone. Indian J. Med. Res..

[176] Cao H., Kuboyama N. (2010). A biodegradable porous composite scaffold of PGA/β-TCP for bone tissue engineering. Bone.

[177] Fabbri P., Cannillo V., Sola A., Dorigato A., Chiellini F. (2010). Highly porous polycaprolactone-45S5 Bioglass^®^ scaffolds for bone tissue engineering. Compos. Sci. Technol..

[178] Abdollahi S., Ma A.C.C., Cerruti M. (2013). Surface transformations of Bioglass 45S5 during scaffold synthesis for bone tissue engineering. Langmuir.

[179] Huang W., Shi X., Ren L., Du C., Wang Y. (2010). PHBV microspheres–PLGA matrix composite scaffold for bone tissue engineering. Biomaterials.

[180] Linh N.T.B., Min Y.K., Lee B.-T. (2013). Fabrication and in vitro evaluations with osteoblast-like MG-63 cells of porous hyaluronic acid-gelatin blend scaffold for bone tissue engineering applications. J. Mater. Sci..

[181] Zhang K.-R., Gao H.-L., Pan X.-F., Zhou P., Xing X., Xu R., Pan Z., Wang S., Zhu Y., Hu B. (2019). Multifunctional bilayer nanocomposite guided bone regeneration membrane. Matter.

[182] Retzepi M., Donos N. (2010). Guided bone regeneration: Biological principle and therapeutic applications. Clin. Oral Implants Res..

[183] Abe G.L., Sasaki J.-I., Katata C., Kohno T., Tsuboi R., Kitagawa H., Imazato S. (2020). Fabrication of novel poly (lactic acid/caprolactone) bilayer membrane for GBR application. Dent. Mater..

[184] Yoshimoto I., Sasaki J.-I., Tsuboi R., Yamaguchi S., Kitagawa H., Imazato S. (2018). Development of layered PLGA membranes for periodontal tissue regeneration. Dent. Mater..

[185] Requicha J.F., Viegas C.A., Hede S., Leonor I.B., Reis R.L., Gomes M.E. (2016). Design and characterization of a biodegradable double-layer scaffold aimed at periodontal tissue-engineering applications. J. Tissue Eng. Regen. Med..

[186] Requicha J.F., Viegas C.A., Muñoz F., Azevedo J.M., Leonor I.B., Reis R.L., Gomes M.E. (2014). A tissue engineering approach for periodontal regeneration based on a biodegradable double-layer scaffold and adipose-derived stem cells. Tissue Eng. Part A.

[187] Park C.H., Rios H.F., Jin Q., Bland M.E., Flanagan C.L., Hollister S.J., Giannobile W.V. (2010). Biomimetic hybrid scaffolds for engineering human tooth-ligament interfaces. Biomaterials.

[188] Eltom A., Zhong G., Muhammad A. (2019). Scaffold Techniques and Designs in Tissue Engineering Functions and Purposes: A Review. Adv. Mater. Sci. Eng..

[189] Aldemir Dikici B., Dikici S., Reilly G.C., MacNeil S., Claeyssens F. (2019). A novel bilayer polycaprolactone membrane for guided bone regeneration: Combining electrospinning and emulsion templating. Materials.

[190] Liu X., Ma P.X. (2004). Polymeric scaffolds for bone tissue engineering. Ann. Biomed. Eng..

[191] Yuan B., Zhou S., Chen X. (2017). Rapid prototyping technology and its application in bone tissue engineering. J. Zhejiang Univ. B.

[192] Leong K.F., Cheah C.M., Chua C.K. (2003). Solid freeform fabrication of three-dimensional scaffolds for engineering replacement tissues and organs. Biomaterials.

[193] Lee J.W., Kim J.Y., Cho D.-W. (2010). Solid free-form fabrication technology and its application to bone tissue engineering. Int. J. Stem Cells.

[194] Stevens B., Yang Y., Mohandas A., Stucker B., Nguyen K.T. (2008). A review of materials, fabrication methods, and strategies used to enhance bone regeneration in engineered bone tissues. J. Biomed. Mater. Res. Part B Appl. Biomater. Off. J. Soc. Biomater. Jpn. Soc. Biomater. Aust. Soc. Biomater. Korean Soc. Biomater..

[195] Ozbolat I.T., Peng W., Ozbolat V. (2016). Application areas of 3D bioprinting. Drug Discov. Today.

[196] Ozbolat I.T., Yu Y. (2013). Bioprinting toward organ fabrication: Challenges and future trends. IEEE Trans. Biomed. Eng..

[197] Datta P., Ozbolat V., Ayan B., Dhawan A., Ozbolat I.T. (2017). Bone tissue bioprinting for craniofacial reconstruction. Biotechnol. Bioeng..

[198] Dehghani F., Annabi N. (2011). Engineering porous scaffolds using gas-based techniques. Curr. Opin. Biotechnol..

[199] Liao C., Chen C., Chen J., Chiang S., Lin Y., Chang K. (2002). Fabrication of porous biodegradable polymer scaffolds using a solvent merging/particulate leaching method. J. Biomed. Mater. Res. Off. J. Soc. Biomater. Jpn. Soc. Biomater. Aust. Soc. Biomater. Korean Soc. Biomater..

[200] Puppi D., Chiellini F., Piras A.M., Chiellini E. (2010). Polymeric materials for bone and cartilage repair. Prog. Polym. Sci..

[201] Liang H.-Q., Wu Q.-Y., Wan L.-S., Huang X.-J., Xu Z.-K. (2013). Polar polymer membranes via thermally induced phase separation using a universal crystallizable diluent. J. Memb. Sci..

[202] Mikos A.G., Temenoff J.S. (2000). Formation of highly porous biodegradable scaffolds for tissue engineering. Electron. J. Biotechnol..

[203] Johnson T., Bahrampourian R., Patel A., Mequanint K. (2010). Fabrication of highly porous tissue-engineering scaffolds using selective spherical porogens. Biomed. Mater. Eng..

[204] Roseti L., Parisi V., Petretta M., Cavallo C., Desando G., Bartolotti I., Grigolo B. (2017). Scaffolds for bone tissue engineering: State of the art and new perspectives. Mater. Sci. Eng. C.

[205] An J., Teoh J.E.M., Suntornnond R., Chua C.K. (2015). Design and 3D printing of scaffolds and tissues. Engineering.

[206] Nair K., Gandhi M., Khalil S., Yan K.C., Marcolongo M., Barbee K., Sun W. (2009). Characterization of cell viability during bioprinting processes. Biotechnol. J. Healthc. Nutr. Technol..

[207] Abdelaal O.A.M., Darwish S.M.H. (2013). Review of rapid prototyping techniques for tissue engineering scaffolds fabrication. Characterization and Development of Biosystems and Biomaterials.

[208] Hutmacher D.W. (2000). Scaffolds in tissue engineering bone and cartilage. The Biomaterials: Silver Jubilee Compendium.

[209] Kumbar S.G., James R., Nukavarapu S.P., Laurencin C.T. (2008). Electrospun nanofiber scaffolds: Engineering soft tissues. Biomed. Mater..

[210] Zafar M., Najeeb S., Khurshid Z., Vazirzadeh M., Zohaib S., Najeeb B., Sefat F. (2016). Potential of electrospun nanofibers for biomedical and dental applications. Materials.

[211] Bikiaris D.N., Papageorgiou G.Z., Achilias D.S., Pavlidou E., Stergiou A. (2007). Miscibility and enzymatic degradation studies of poly (ε-caprolactone)/poly (propylene succinate) blends. Eur. Polym. J..

[212] Ramakrishna S., Fujihara K., Teo W.-E., Yong T., Ma Z., Ramaseshan R. (2006). Electrospun nanofibers: Solving global issues. Mater. Today.

[213] Jang J.-H., Castano O., Kim H.-W. (2009). Electrospun materials as potential platforms for bone tissue engineering. Adv. Drug Deliv. Rev..

[214] Agarwal S., Wendorff J.H., Greiner A. (2009). Progress in the field of electrospinning for tissue engineering applications. Adv. Mater..

[215] Jiang T., Carbone E.J., Lo K.W.-H., Laurencin C.T. (2015). Electrospinning of polymer nanofibers for tissue regeneration. Prog. Polym. Sci..

[216] Hussain M., Ullah S., Raza M.R., Abbas N., Ali A. (2022). Recent Developments in Zn-Based Biodegradable Materials for Biomedical Applications. J. Funct. Biomater..

[217] Howard D., Buttery L.D., Shakesheff K.M., Roberts S.J. (2008). Tissue engineering: Strategies, stem cells and scaffolds. J. Anat..

