# Correction: Alqahtani et al. Guided Tissue and Bone Regeneration Membranes: A Review of Biomaterials and Techniques for Periodontal Treatments. *Polymers* 2023, *15*, 3355

**DOI:** 10.3390/polym17070863

**Published:** 2025-03-24

**Authors:** Ali M. Alqahtani, Robert Moorehead, Ilida Ortega Asencio

**Affiliations:** 1 Department of Restorative Dental Sciences, College of Dentistry, King Khalid University, Al Fara, Abha 62223, Saudi Arabia; aqahtani@kku.edu.sa; 2 The Department of Materials Science and Engineering, The University of Sheffield, Sir Robert Hadfield Building, Sheffield S1 3JD, UK; r.moorehead@sheffield.ac.uk; 3 The School of Clinical Dentistry, The University of Sheffield, 19 Claremont Crescent, Sheffield S10 2TA, UK

Dr. Robert Moorehead and Dr. Ilida Ortega Asencio were not included as authors in the original publication [1]. The corrected Author Contributions Statement appears here.

**Author Contributions:** A.M.A.: Conceptualization, Methodology, Validation, Formal Analysis, Investigation, Resources, Data Curation, Writing—Original Draft, Writing—Review and Editing, Visualization, Supervision, Project Administration, Funding Acquisition. R.M.: Conceptualization, Methodology, Supervision, Validation, Visualization, Writing—Review and Editing. I.O.A.: Conceptualization, Methodology, Supervision, Validation, Visualization, Writing—Review and Editing. All authors have read and agreed to the published version of the manuscript.

The authors apologize for any inconvenience caused and state that the scientific conclusions are unaffected. This correction was approved by the Academic Editor. The original publication has also been updated.
